# Delivery and evaluation of participatory education for animal keepers led by veterinarians and para-veterinarians around the Kanha Tiger Reserve, Madhya Pradesh, India

**DOI:** 10.1371/journal.pone.0200999

**Published:** 2018-08-02

**Authors:** Andy Hopker, Naveen Pandey, Aniruddha Dhamorikar, Sophie Hopker, Pradeep Gautam, Subash Pandey, Sharad Kumar, Narendra Rahangadale, Prakash Mehta, Rebecca Marsland, Neil Sargison

**Affiliations:** 1 The Royal (Dick) School of Veterinary Studies, The University of Edinburgh, Easter Bush Veterinary Centre, Midlothian, Scotland, United Kingdom; 2 The Corbett Foundation, Baherakhar, near Kanha Tiger Reserve, Madhya Pradesh, India; 3 School of Social and Political Sciences, The University of Edinburgh, George Square, Edinburgh, Scotland, United Kingdom; B P Koirala Institute of Health Sciences, NEPAL

## Abstract

**Aim:**

We aim to investigate local perceptions of animal health challenges; current animal health knowledge; and methods to provide effective, relevant education to animal keepers in the Kanha Tiger Reserve area.

**Materials and methods:**

A farmer education programme was undertaken in the Kanha Tiger Reserve area. Local animal health priorities were investigated through participatory village meetings (n = 38), individual animal keeper questionnaires (n = 100) and a written survey of local paravets (n = 16). Educational interventions were: veterinary surgeon led education meeting (VE); paravet led education meeting (PVE); distribution of printed materials (PM). 230 village meetings were carried out across 181 villages, contacting 3791 animal keepers. 20 villages received printed materials. Information was gathered on perceptions of local animal health challenges and current remedies. Efficacy of knowledge transfer was assessed four to five months later using a purposeful sample of 38 villages.

**Results:**

Group meetings identified ticks (35/38), foot and mouth disease (FMD) (31/38) and diarrhoea (30/38) as the greatest animal health challenges. Individual interviews identified haemorrhagic septicaemia (HS) (87/100), blackquarter (BQ) (66/100) and plastic ingestion (31/100). Paravets identified FMD (7/16), BQ (6/16) and HS (6/16), and also indicated that animal husbandry and socio-economic factors were important. Current treatments were primarily home remedies and herbalism, but also included contacting a paravet, use of pharmaceuticals and faith healing. Animal treatment knowledge prior to intervention was not significantly different between groups (P = 0.868). Following intervention animal health knowledge was assessed: PVE performed better than controls (P = 0.001) and PM (P = 0.003); VE performed better than controls (P = 0.009). There was no significant difference between VE and PVE (P = 0.666) nor PM and controls (P = 0.060).

**Conclusions and recommendations:**

Open access participatory village meetings are an effective way to provide animal health education. In this region distribution of posters and leaflets did not appear to be an effective way to contact animal keepers. Meetings led by paravets can be as effective as those led by veterinarians and paravets can rapidly and sustainably contact large numbers of animal keepers. Investigation of the local animal health situation is essential to ensure education is relevant and accessible to intended recipients. Interventions must be carefully planned to maximise engagement of all sections of the community, particularly women.

## Introduction

Improved livestock health, welfare and productivity is a route to improved food security and prosperity for rural people, and the use of efficient farming techniques can help to protect the natural environment and reduce human- wildlife conflict. Community education of animal keepers has a role in achieving these goals sustainably.

The majority of farmers in India live and work on small family holdings in traditional rural communities. There are 263 million people employed in agriculture in India [1] and the average holding occupies an area of just 1.16 hectares [2]. The high input of manual labour combined with the limited potential to derive profit from such small enterprises means that it is difficult for these farmers to build infrastructure or invest for the future. Consequently the agricultural sector contributes 17% of India’s Gross Domestic Product despite employing 49% of the country’s workforce [3]. Women carry out more than half of all agricultural work in India [4] and seventy eight percent of rural female workers are employed in agriculture, mainly as unpaid workers on family farms or as casual labourers, and so their involvement in animal husbandry is not fully captured by the Indian Census [5].

Cattle are central to the livelihoods of Indian villagers. The main source of agricultural power is draft bullocks and manure is used both as agricultural fertiliser and fuel, and crops are grown for home consumption and sale. Milk from cows and goats provides essential protein for households and well- organised dairying co-operatives with milk collection services make it possible for very small scale producers to sell milk. Breeding and sale of animals, when possible, provides additional cash income. Cattle also have an important social and spiritual role in Indian village communities. Co-operatives are helping small producers to work together more efficiently, however there is a need for farmer education at a local level to improve standards of animal husbandry and health, thereby improving levels of productivity and welfare.

Livestock grazing exerts tremendous pressure on the forests [6] resulting in competition for resources with wild herbivores as well as reducing the natural regeneration capacity of the forests. Regular incursions into forests for livestock grazing also increases chances of livestock depredation by wild carnivores such as tigers [7], while at the same time also increases the potential of transferring diseases from domestic to wild animals [8]. Introduction of more efficient farming and animal husbandry techniques has the potential to reduce resource competition, forest penetration, habitat degradation, and human wildlife conflict, helping in preserving the natural ecosystems [9].

Previous studies involving farmer focused knowledge transfer programmes in Africa have found measurable improvements in animal health understanding following interventions [10, 11, 12]. Knowledge exchange programmes must reach the target audience, be relevant to the local situation and be accessible, acceptable, and understandable, so that the knowledge be retained. Visual aids can be of benefit to an education programme, however the ability of the reader to interpret this material, referred to as “visual literacy” must be considered, and the material piloted to ensure comprehension [13, 14].

Rural education programmes must engage all sections of the rural community. Women are key workers in the agricultural sector in India, at the same time as manging complex households and often pursuing additional livelihood strategies [15]. Engaging rural women in education programmes is more challenging than contacting their male contemporaries [16]. Scheduled Tribal (ST) people, also referred to as Adivasi, are distinct indigenous ethnic communities in India. ST people are considered vulnerable and offered some protections under Indian law and ST communities may be particularly sensitive to ecological disturbance or inward migration. ST people make up make up 8.6% of the population of India but 21.1% of the population of Madhya Pradesh, and tend to concentrate in rural, particularly forested, areas [1, 17]. Bagia and Gond peoples are the main tribes found in the KTR. Poor levels of literacy are a potential challenge for any rural education programme, the overall literacy rate in rural Madhya Pradesh is 64%, however for ST people it quoted as 41.2% overall [1] or 52.5% for men and 27.2% for women [18]. As an important section of the rural community, special efforts should be made to ensure the inclusion of ST people in any animal keeper education project.

Farmer education has the potential to improve human and animal health and wellbeing through promoting efficient, welfare- friendly production and this sustainable use of resources benefits both human populations and the natural environment. Investigation into effective means to provide this education helps to drive forward rural development goals.

## Materials and methods

### Ethical approval

The work was carried out under ethical approval from the R(D)SVS University of Edinburgh Veterinary Ethical Review Committee (VERC approval number 74 14) and by agreement with the Panchayats (village councils) arranged by The Corbett Foundation. The aims of the project and the use of information gathered, including photographs, in potential publications was explained to the participants, in Hindi and local dialects when necessary, with participants having the opportunity to ask questions about the process throughout. Participants gave verbal consent for participation in the study, the use of photography and the use of all study material, including photographs, for publication. Written permission was not taken due to the level of illiteracy in the area and the local cultural mistrust of signed documents, which would reduce participation in the education programme. This methodology was approved by the R(D)SVS ethical review committee.

### Study area

**Fig 1:**
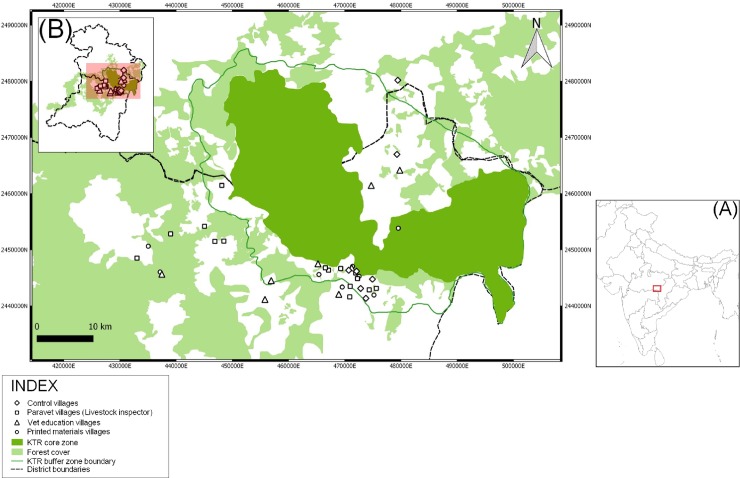
Map of the Kanha Tiger Reserve (KTR) Core and Buffer Zones, showing villages visited for assessment of knowledge transfer. Symbol denotes previous intervention. (A) Map of India showing the location of the Kanha Tiger Reserve within the state of Madhya Pradesh. (B) Study area shown within the districts of Mandla and Balaghat. (A. Dhamorikar).

Kanha Tiger Reserve (KTR) was one of the first Indian Tiger Reserves declared in 1973 under Project Tiger. It is located in the state of Madhya Pradesh, and lies within the districts of Mandla and Balaghat (Fig 1). 181 villages are present in and around KTR (161 in a peripheral Buffer zone and 20 in the central Core Zone). Human activity within the core region is strictly limited and grazing of livestock only permitted for the few settlements found within the core zone. Movement into the Core zone of the KTR is closely controlled by a permit system. The Buffer zone can however be travelled relatively freely and grazing of livestock is widespread. The primary enterprises in the settlements of the KTR are agriculture and animal husbandry, draft bullocks provide the main source of power for agricultural work. Veterinary care for livestock available to animal keepers of the KTR is mostly provided by local paravets known as ‘Gau Sewek’ (literally translated as ‘cow carers’, also called ‘Livestock Inspectors’). These paravets mostly operate as small private enterprises paid directly by the animal keepers, though some are employed by The Corbett Foundation (an Indian NGO) and charge a nominal fee for their work. To our knowledge there are no other NGOs providing veterinary services for livestock in the area. Government veterinary services are available from the veterinary hospitals in Baihar and Mohgaon, however these services are under- utilised by the respondents because of the cost and distance.

### Participants

Respondents in this study were animal keepers of the villages of the KTR and local paravets (Gau Sewek). A purposeful sample of villages was used to include a representative selection of local conditions and ethnic groups. Every effort was made to include all sections of the rural community, village meetings were open access, and all who wished to attend could to do so free of charge. Para- vets were recruited into the programme by making a personal invitation to all qualified Gau Sewek practicing in the KTR. Meetings were facilitated by staff from The University of Edinburgh, Scotland and The Corbett Foundation, India.

### Study design

Knowledge transfer activities for animal keepers on the subject of improving on animal health, welfare and productivity were carried out in villages of the KTR. Prior to knowledge transfer, information was collected from participants through open group questions during village meetings, regarding pre-existing attitudes to animal health challenges and current practices in animal husbandry and treatments. The efficacy of knowledge transfer was assessed four months later using a semi- structured group interviews during participatory educational village meetings. The three different methods of knowledge transfer used are described below.

#### i) Veterinary surgeon led educational meetings (Vet Education/ VE)

**Fig 2:**
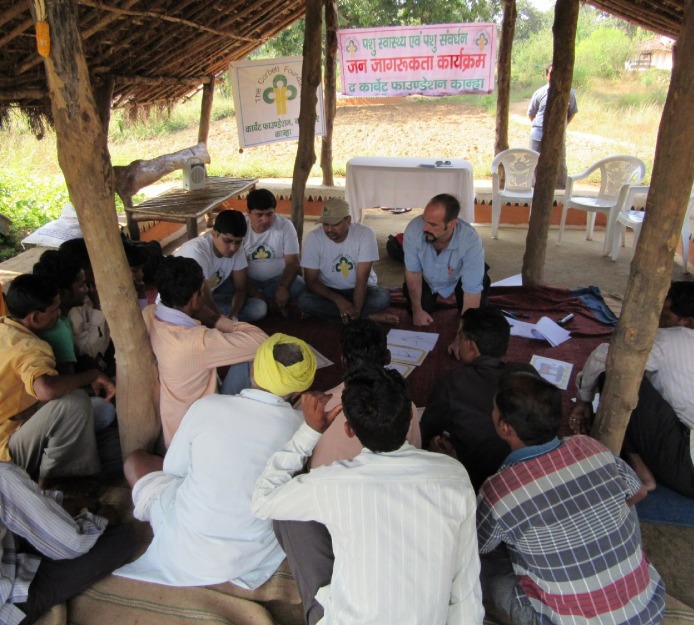
Participatory village meeting conducted outdoors. Participants discuss animal health concerns with each other and the study team. Men and women have chosen to sit separately.

Nine villages received participatory educational meetings facilitated by a veterinary team. Participants discussed their animal health concerns and simple hand drawn pictures illustrating animal health problems were then used to further stimulate discussion on animal health challenges in a participatory fashion. Facilitators and animal keepers sat in a circle on the floor and animal keepers were encouraged to share and discuss their experiences (Fig 2). Information was collected on current animal husbandry practices followed by the respondents, including treatment of their animals. The meetings followed a semi- structured format and were flexible enough to address the needs and questions of each group. During the meeting an individual animal health poster was produced for display in the village using the same hand drawn images from the meeting and Hindi text. All attendees were presented with a set of measuring spoons with attached instructions in cartoon format and Hindi text for use in making oral rehydration solution (ORS) for the treatment of diarrhoea in animals and children. The educational team for all meetings included an Indian vet, a UK vet and a local para- vet. These nine meetings contacted 181 animal keepers.

#### ii) Printed educational materials (Print/ PM)

20 villages received printed educational materials. Each village had two posters displayed in prominent public places. Leaflets and measuring spoon sets with instructions were handed directly to ten animal keepers in each village by a local paravet. Printed materials were based around the same images used in the VE meetings and imparted messages in a cartoon style in order to contact non- readers, but brief text descriptions in Hindi were also included. Five animal keepers in each village were individually interviewed by a local paravet prior to receiving the leaflets. The brief structured interview included listing their top 3 animal health challenges and current treatment practices for wounds, diarrhoea and pneumonia (S1 Text). No further knowledge transfer was provided at that time.

#### iii) Paravet (Gau Sewak) led educational meetings (Paravet Education / PVE)

The experience gained conducting meetings and producing printed materials was employed to design and produce an education pack for use by paravets or animal health workers to conduct educational meetings. This pack utilised a combination of photographic images, cartoon drawings and Hindi text. The pack was fully laminated for durability and included guidance notes in Hindi on conducting educational village meetings. Eighteen of the 21 qualified paravets practicing in the region wished to participate in the programme and attended one day of training. During the training day paravets completed questionnaires (S2 Text) about their attitudes regarding challenges to animal health and productivity. The paravets were issued with booklets to record the meetings taken and collect animal health information from each village group. The para- vets used the education packs to conduct a series of educational participatory meetings in the region where they normally work. All the villages of the KTR (181 villages) received a participatory educational meeting led by their local para- vet, 2893 animal keepers were contacted over a period of 10 weeks.

### Assessment of knowledge transfer

Efficacy of knowledge transfer activities was assessed through a further series of veterinary surgeon led participatory village meetings following a semi-structured format. These meetings were carried out between four and five months after the initial knowledge transfer activities. Thirty eight village meetings were included in the study (Fig 1). These were as follows: seven VE villages; eight PM villages; 16 PVE villages; and 7 control villages (C). The group of villages referred to as the control group (C) had not received a previous intervention as part of this study. Villages were selected as a purposeful sample to allow a fair representation of local ethnic groups and regional conditions. One village previously visited by each of the 16 participating paravets was revisited. The decision to visit the region of each participating paravet was taken to maximise their continuing engagement and avoid offending any paravets whose region did not receive a re-visit. Only PVE villages which had received no other intervention (PM, VE or C) were selected for re-visits. Meetings were open to all who wished to attend.

**Fig 3:**
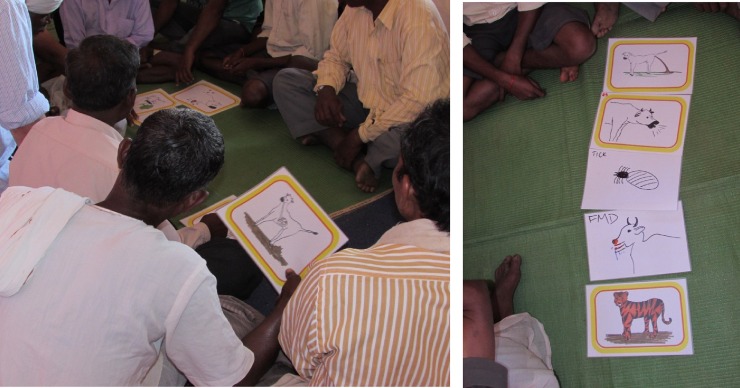
Participative ranking. Participants select animal health challenges of concern to them and then work as a group to rank them and indicate relevant topics for further discussion.

The meetings commenced with animal keepers discussing their animal health concerns, selecting the issues of greatest concern to themselves, and then working as a group to produce a “top five” list of animal health concerns by participative ranking [19], shown in Fig 3. The remainder of the meeting covered eight topics: FMD, maggot wounds (myiasis), diarrhoea, bovine respiratory disease (BRD), housing and husbandry, *Lantana camara* (an invasive plant causing toxicity and photosensitisation) ingestion, intestinal worms and zoonotic spread of disease. These issues were selected based on the findings of the initial vet led meetings where participants were free to discuss any topics they wished with the veterinary team. Colour photographs and hand drawn images were used to stimulate discussion of each topic (S1 Fig) and a series of repeatable accompanying questions were posed to every meeting group (S3 and S4 Texts). These questions were based on the categories: identification of the problem; causes of the problem; treatment; and prevention. The group discussed the topic until a consensus or prevailing opinion emerged. On occasions where no consensus emerged the group was asked to vote by show of hands. Responses were awarded one mark for a good answer, for example: correct identification of a problem from an image; demonstration of accurate understanding of cause; discussion of use of an effective treatment or preventative measure. A half mark was awarded for a partial answer such as: identification of a similar but different problem; a slightly confused understanding of cause; a treatment regime missing some key elements, or a suboptimal preventative strategy in the local circumstances. A half mark was also awarded if a group initially produced no answer and then subsequently gave a good response following prompting from the meeting facilitators. No marks were awarded if the answer was poor, directly injurious to the animal; or no response was offered. The maximum total score for the exercise was 36. Answers were recorded and marks were assigned by a primary marker and moderated by other team members to ensure consistency of marking. The meetings were designed to have inherent educational value to the participants, however knowledge transfer from the team to the participants was not carried out until after the assessment phase of the discussion of each topic. The meeting team were careful to be consistent in approach and to avoid introducing bias through asking leading questions.

Between November 2014 and April 2016, a total of 230 village meetings were carried out across the 181 villages of the KTR. 3791 animal keepers were contacted and there was an overall female participation rate of 27% (1023/ 3791). Participants typically had between 2 and 4 adult cattle or buffalo in addition to a small number of goats and chickens. 20 villages received printed materials. (Table 1).

**Table 1:** Knowledge transfer interventions undertaken as part of the programme.

	11/14	3/15	3/15–4/15	11/15	11/15–1/16	4/16
Intervention	VE	VE	PM	VE	PVE	VE
Number of villages	9	16	20	8	181	16
Number of participants	181	296	200+	155	2893	266
Female participation	10%	21%	?	18%	30%	15%

The total number of interventions, 250, is greater than the 181 villages in the study area. This is because villages included in the study received two interventions as they were revisited for assessment and all villages in the KTR received a PVE regardless of any previous interventions as part of this project. Villages which received both PVE and another intervention were not included in the study. 200 animal keepers were handed a leaflet, however the actual number of people reading leaflets is unknown.

### Data analysis

Data was analysed using Minitab 17 ^TM^ (Minitab Inc.) and R version 3.3.1 (The R Foundation) software. Information about the animal treatment knowledge of participants of the C, VE and PM groups prior to intervention was gathered through three open questions about the treatment of three common conditions (diarrhoea, respiratory disease and maggot wounds). Each topic was marked on a four point scale. Because the data is on a ranking scale the non- parametric equivalent to a one- way ANOVA, the Kruskal- Wallis test by ranks was used to determine whether the groups had different animal treatment knowledge prior to receiving knowledge transfer through the programme. Similar pre- intervention data is not available for the PVE group. The significance of post- intervention results was determined using the Kruskal- Wallis test by ranks followed by Dwass, Steel, Critchlow- Fligner non- parametric pair-wise post-hoc testing to investigate the effect of knowledge transfer technique on animal health knowledge. The assessment results were examined as overall (total) scores; by topic (condition); and by question type (identification of problem, causes of disease, treatment and prevention). The Dwass, Steel, Critchlow- Fligner two sided all treatments comparison procedure allows the simultaneous comparison of the means of all pairs of groups while simultaneously controlling the error rate [20]. P values of < 0.05 were taken to indicate statistical significance and degrees of freedom for the Kruskal Wallis test are shown as subscripts.

## Results

Following knowledge transfer interventions thirty eight village meetings were carried out with the purpose of assessing efficacy of knowledge transfer (Table 2).

**Table 2:** Village meetings used for assessment of knowledge transfer and mean overall scores.

Group	Number of village meetings	Participants			Mean group score (-/36)
		Total	Male	Female	
Control (C)	7	170	118 (66%)	52 (34%)	19.5 (54%)
Printed Materials (PM)	8	155	127 (82%)	28 (18%)	23 (64%)
Vet Education (VE)	7	124	115 (93%)	9 (7%)	30 (83%)
Paravet Education (PVE)	16	266	226 (85%)	40 (15%)	28 (78%)

### Perceived animal health challenges

**Fig 4:**
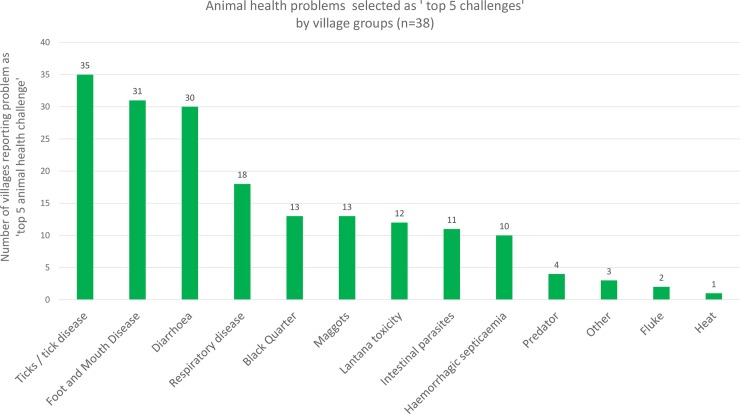
Major animal health challenges as agreed by participant groups at village meetings.

**Fig 5:**
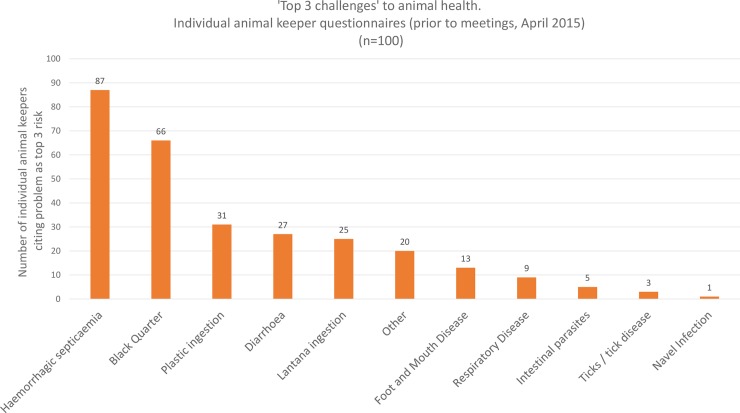
Animal health challenges as selected by individual animal keepers questioned alone.

**Fig 6:**
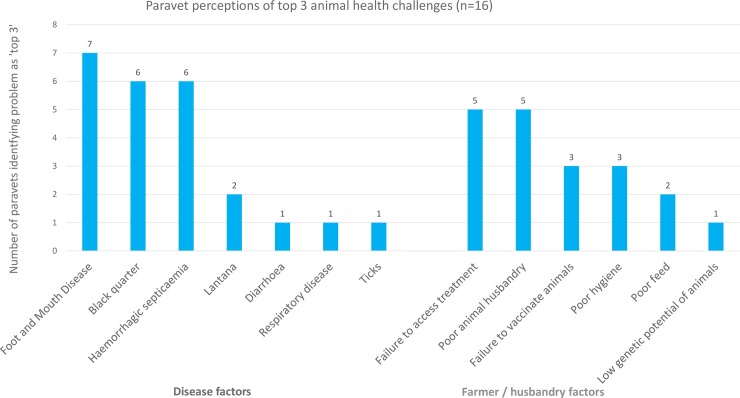
Paravet attitudes to animal health challenges.

Top five animal health challenges as selected by village meeting groups are shown in Fig 4. Group meetings most frequently selected ticks and tick born disease as a top five animal health challenge (35/38 groups). This was followed by foot and mouth disease (FMD) (31/38); diarrhoea (30/38); bovine respiratory disease (BRD) (18/38); Blackquarter (BQ) (13/38) and maggots (13/38). Three groups also described a local condition, characterised by the formation of a single blood filled structure (haematoma?) beneath the tongue, as a major problem. All meeting groups discussed this condition. Top three animal health challenges selected by individual animal keepers prior to knowledge transfer are shown in Fig 5. Individuals most frequently selected haemorrhagic septicaemia (HS) as a top three animal health challenge (87/100); followed by BQ (66/100); plastic ingestion (31/100); diarrhoea (27/100) and lantana ingestion (25/100). 20 individuals listed the sublingual haematoma described above as a top three challenge. Perceptions of top three animal health challenges by paravets are shown in Fig 6. Local paravets rated HS (7/16); BQ (6/16) and FMD (6/16) as the top three animal health challenges. Paravets identified various farmer and husbandry factors as being important, though this subject was not raised by farmer groups. Local animal health terms used in the KTR region can be found in (S1 Table).

Vaccination as a means of preventing disease was discussed by participants without prompting in 35/38 village meetings, though no meeting group reported 100% vaccine usage. Some or all participants in 37/38 meetings reported that they deliberately withheld colostrum from neonatal calves, or considerably limited colostrum intake, in the belief that this practice would reduce the occurrence of diarrhoea.

Predation by tigers (*Panthera tigris*) and leopards (*Panthera pardus fusca*) were reported as a major risk in two villages, both of which were located immediately adjacent to the Core Zone. Tail tip necrosis was discussed at most meetings, participants reported it to be prevalent during and immediately after the monsoon. Following necrosis, part affected portion of the animal’s tail sloughs to leave an open wound.

### Treatments in current use in the KTR

Currently used remedies discussed by village meeting participants for FMD, maggot wounds, diarrhoea, BRD and intestinal parasites are shown in Table 3.

**Table 3:** Treatments reported to be currently in use by animal keepers in the KTR. All treatments reported by three or more groups are shown.

Condition	Treatment	Number of groups reporting use (-/38)
Diarrhoea	**Maida bark infusion *(Litsea Glutinosa)***	24 (63%)
	**Rice husk**	10 (26%)
	**Drinking water**	7 (18%)
	**Salt/ sugar solution (homemade ORS)**	6 (16%)
	**Call paravet**	6 (16%)
	**Multaina (Clay soil)**	5 (13%)
	**Rice water**	5 (13%)
	**Neem leaves (*Azadirachta indica)***	5 (13%)
	**Hing (asafoetida) (*Ferula assa-foetida*)**	3 (8%)
	**Butter milk**	3 (8%)
Respiratory disease	**Call paravet**	27 (71%)
	**Infusion of tea, spices and jaggery**	16 (42%)
	**Drink of jaggery, honey and sugar**	7 (18%)
	**Rest**	6 (16%)
	**Mushrooms (not specified)**	4 (11%)
	**Methi (fenugreek) (*Trigonella foenum-graecum***)	3 (8%)
	**Haldi (turmeric) (*Curcuma longa*)**	3 (8%)
	**No treatment**	3 (8%)
FMD	**Coriander leaf (*Coriandrum sativum*) compress (tongue)**	21 (55%)
	**Mud application/ compress (feet)**	17 (45%)
	**Faith healing / witchcraft**	11 (29%)
	**Salt compress (tongue)**	11 (29%)
	**Phenyl disinfectant application**	6 (16%)
	**Himax**^**TM**^	4 (11%)
	**Petrol/ kerosene application**	4 (11%)
	**Napthalene (Moth balls)**	3 (8%)
	**Camphor *(Cinnamomum camphora)***	3 (8%)
	**Aggressive debridement**	3 (8%)
	**Mustard oil**	3 (8%)
Maggot wounds	**Wound cleaning and maggot removal**	20 (53%)
	**Napthalene (moth balls)**	13 (34%)
	**Phyenolic disinfectant**	11 (29%)
	**Petrol / Kerosene application**	10 (26%)
	**Haldi (turmeric) (*Curcuma longa*)**	10 (26%)
	**Deltamethrin**	7 (18%)
	**Custard apple leaf compress *(Annona reticulate)***	6 (16%)
	**Herbal compress (not specified)**	5 (13%)
	**Camphor *(Cinnamomum camphora)***	4 (11%)
	**Himax**^**TM**^	4 (11%)
	**Tobacco**	4 (11%)
	**Neem (*Azadirachta indica)***	3 (8%)
	**Mustard oil**	3 (8%)
**Intestinal nematodes**	**Neem (*Azadirachta indica)***	14 (37%)
	**Antihelminthic drug (not specified)**	10 (26%)
	**Call paravet**	10 (26%)
	**Piperazine**	7 (18%)
	**Indrayan (*Citrullus colocynthis) (*Bitter thing)**	6 (16%)
	**Bamboo leaf**	5 (13%)
	**Karonda leaf or fruit** (***Carissa carandas)***	4 (11%)
	**Albendazole**	3 (8%)
	**Hing (asafoetida) (*Ferula assa-foetida*)**	3 (8%)
	**Buttermilk**	3 (8%)

For full lists of all treatments reported in use, please see supplementary information (S2–S4 Figs.).

### Assessment of knowledge transfer

**Fig 7:**
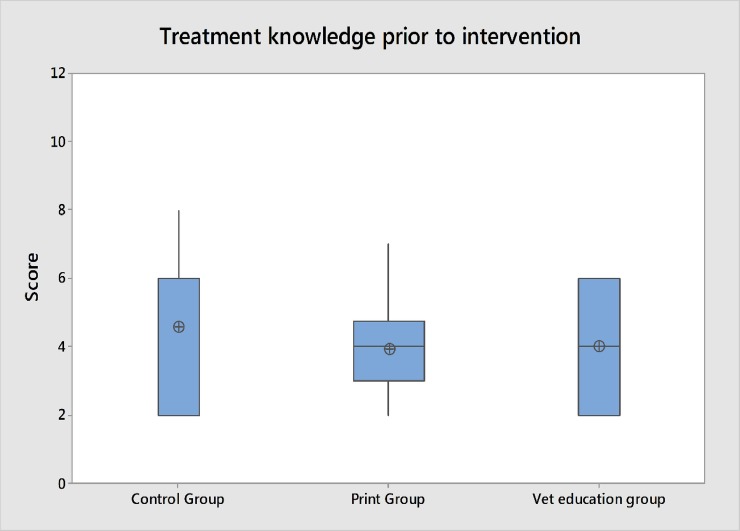
Animal treatment knowledge scores prior to knowledge transfer. Scores generated using standard questions at village meetings.

Animal treatment knowledge scores prior to intervention are shown in Fig 7. Use of the Kruskal- Wallis test by ranks shows that there is no significant difference between different groups knowledge of animal treatment prior to knowledge transfer activities(P = 0.868) (Table 4), indicating that these villages had broadly similar levels of animal health knowledge prior to intervention.

**Table 4:** Comparison of assessed group meeting knowledge pre and post intervention.

	Median (interquartile range)	Kruskal- Wallis	Post-hoc pairwise comparison (Dwass, Steel, Critchlow- Fligner)
**Pre- intervention** **(score: -/12)**	C: 6 (IQR = 4) PM: 4 (ICR = 1.75) VE: 4 (IQR = 4)	**H_2_ = 0.28** **P = 0.868**	N/A
**Post- intervention** **(score: -/36)**	C: 20 (IQR = 3) PM: 23.75 (IQR = 4.5) VE: 32 (IQR = 9.5) PVE: 28.5 (IQR = 3.5)	**H_3_ = 23.9** **P < 0.001**	C vs PM, P = 0.060 C vs VE, P = 0.009* C vs PVE, P = 0.001* PM vs VE, P = 0.107 PM vs PVE, P = 0.003* VE vs PVE, P = 0.666

Kruskal- Wallis table shows significance of variation in animal health knowledge between groups before and after knowledge transfer. Degrees of freedom are shown as subscripts. Dwass, Steel, Critchlow- Fligner result shows the significance of variation of assessment scores when comparing groups (C, PM, VE, PVE).

*indicates significant difference

**Fig 8:**
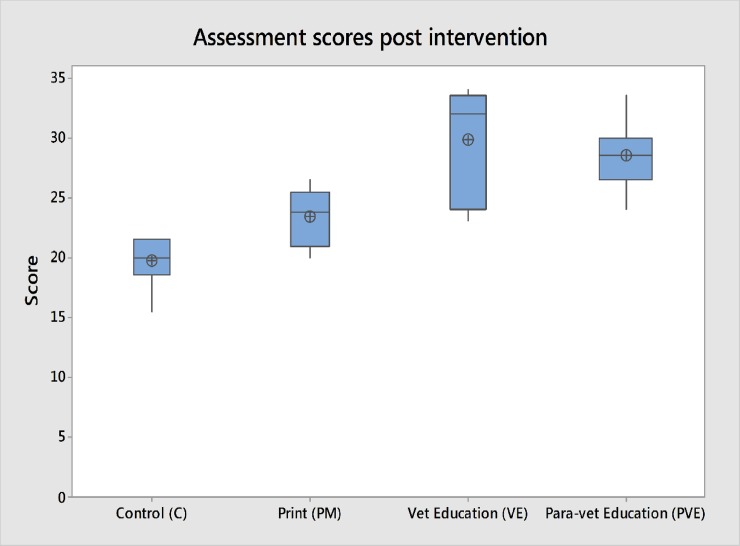
Animal health knowledge of groups following knowledge transfer. Assessment through standard group questions at village meetings. Total scores shown.

Following knowledge transfer there were significant differences in animal health knowledge between groups (Table 4, Fig 8.). PVE groups performed better than C (P = 0.001) and PM groups (P = 0.003). VE groups performed better than controls (P = 0.009). There was no significant difference between VE and PVM groups (P = 0.666), however there was a tendency for VE groups to score more highly than PVE groups at assessed meetings. There was also no significant difference between PM and C groups (P = 0.060) (Dwass, Steel, Critchlow- Fligner non- parametric pair-wise post-hoc testing). A full list of all assessed meeting scores, including breakdown by topic, can be found in Supplementary information (S2 Table).

On examining the groups post intervention knowledge by individual topics it can be seen that VE groups performed significantly better than C on FMD (P = 0.046), diarrhoea (P = 0.016) and pneumonia (P = 0.048). PVE groups performed significantly better than controls on FMD (P<0.001), diarrhoea (P = 0.005), animal housing and husbandry (P = 0.017), lantana toxicity (P = 0.023) and zoonotic disease (P = 0.007). VE groups performed significantly better than P groups on diarrhoea (P = 0.016). PVE groups performed significantly better than PM on FMD (P<0.001) and diarrhoea (P = 0.019). The topics of maggot wounds and intestinal worms produced no significantly different results between any of the groups. For full tables and graphs please refer to supplementary information (S3 Table and S5 Fig.)

Grouping of the same questions by type instead of topic, gave the categories: identification of the problem; causes of disease; treatment; and prevention of the problem (S4 Table and S6 Fig). Identification of problems: VE performed significantly better than C (P = 0.025); PVE performed significantly better than C (P<0.001) and PM (= 0.022). Causes of disease: VE performed significantly better than PVE (P = 0.026) and PM (P = 0.020). Treatment of problems: VE performed significantly better than C (P = 0.035); PVE performed significantly better than C (P = 0.004) and PM (P = 0.049). Prevention of problems: PVE performed significantly better than C (P = 0.021) and PM (P = 0.037).

## Discussion

### Perceived animal health risks

There is considerable variation between the results of the group interviews and those conducted individually (Figs 4 and 5). Group meetings highlighted common diseases, while individuals concentrated on disease fears. FMD and tick borne diseases are endemic in the area, diarrhoea is a common problem. Losses due to these conditions are substantial [21, 22, 23, 24], these conditions were prioritised by meeting groups Individual interviewees highlight BQ and HS, sporadic but highly fatal diseases, and plastic impaction, an infrequent problem in the area due to comparatively low levels of plastic litter. This can be interpreted as individuals stating the diseases which worry them the most, rather than those with the greatest overall impact within a normal year. This difference may be a result of group discussion evaluating the impact on the community and moderating priorities accordingly [25]. This demonstrates the value of participatory methods in establishing local development goals.

Animal keeper meeting groups commonly stated that ticks and tick borne disease are the greatest risk to their cattle, though they did not always interpret disease and vector to be separate entities. Villages in areas where farm animals are commonly free grazed inside the forest tended to consider tick disease to be of greater importance than those villages in areas where no forest grazing is available. Groups often discussed at length increased risk of infestation among forest grazed cattle and advocated keeping prized animals at their dwellings, carrying forage to these animals, despite the increased labour required. Paravets discussed babesiosis and theileriosis as widespread in the region and the team examined clinical cases of these diseases during the study, though because of the variable symptoms and similarity to other conditions, animal keepers may not always associate ill thrift or death caused by these diseases with tick infestation.

The visible nature and widespread occurrence of ticks led participants to assign high importance to them, which may be greater than their impact on cattle health. For example, one village had suffered a recent outbreak of HS resulting in the death of numerous animals, however the ensuing discussion ultimately concluded that ticks were the greater problem as only some of the animals died whereas all of the cattle were infested with ticks. In some cases an individual proposed the last fatal condition which occurred to one of their animals as the most important, often initiating lively debate with fellow group members.

Awareness of the benefits of vaccination for protection from infectious diseases (FMD, HS, BQ) was generally very good, though there was frequently confusion regarding which diseases can be protected against, which vaccinations had been administered and the required frequency of booster vaccinations. Three villages, mainly populated by ST people, did not discuss vaccination, all these villages also reported FMD to be a major problem. Vaccination usage did not reach 100% in any village, the most common reasons discussed by participants were being too busy to present the cattle, and lack of awareness that the vaccination camp was occurring. Only one participant expressed a mistrust of vaccination. Witchcraft and faith healing were discussed by the participants in some villages as being useful for prevention and treatment of FMD. Informal discussion with villagers indicated that witchcraft and faith healing are used more widely than was reported during meetings, but are seldom discussed.

Diarrhoea was commonly discussed as a problem by meeting groups. Participants frequently proposed consumption of dirty water or overconsumption of colostrum by calves as major risk factors for diarrhoea. Only a few individuals recognised the importance of consumption of adequate colostrum shortly after birth in the prevention of neonatal diseases or the occurrence of diarrhoea as an infectious process rather than one of dietary origin. Colostrum was frequently consumed by households, often made into sweets, contributing to limited colostrum supply for calves. Participants were amenable to discussing new concepts such as infectious causes of diarrhoea, the importance of hygiene, colostrum management, and isolation of clinically affected animals in the control of this condition. However reducing colostrum consumption by households may be more challenging as this impacts on local cultural beliefs. Some cases of calf diarrhoea were treated by the team following meetings.

Village groups reported maggot wounds to be an important problem while individual interviewees did not. The team treated maggot wound cases following some meetings. Groups often discussed BRD but tended to consider it a common but minor to moderate problem. BRD was discussed as having varying severity, serious cases requiring para- veterinary attention while mild cases can be treated at home with herbal preparations. Some groups discussed respiratory infections spreading between animals and there was some discussion of the role of air quality in the occurrence of respiratory disease, however few groups linked this to poor ventilation or overcrowding in cattle accommodation.

Meeting groups often discussed lantana ingestion as an important challenge to animal health and productivity, blaming the widespread growth of the plant and a lack of other green forage, particularly during the dry season. Animal keepers frequently reported weakness or jaundice as a problem, these vague clinical signs may result from lantana toxicity, tick borne diseases, or a host of other conditions. Lantana toxicity may present as an acute or delayed condition, which may further increase confusion as can the effect of stall feeding in reducing both lantana ingestion and contact with ticks. It was also discussed that occasionally animals develop a preference for eating lantana. Only two village groups discussed the role of sunlight in the occurrence of skin lesions in lantana toxicity. These groups went on to discuss the benefit of housing affected animals in the shade. Cases of jaundice, weakness and photosensitisation were examined during the study.

The commonly reported method of detection of gastro- intestinal parasites was observation of worms in faeces. Some owners reported deworming their animals on suspicion of intestinal parasites based on ill thrift or poor hair coat. A few owners dewormed calves on a three weekly basis. No participant suggested submitting a faecal sample to a veterinarian or animal health worker for diagnostic examination. This suggests that the prevalence of infestation with gastro- intestinal parasites and their effect on productivity is underestimated by participants.

No group reported umbilical infection of calves as a risk and the drawing frequently had to be explained to participants. This did not prompt any discussion of neonatal umbilical treatment among participants, suggesting that this technique is not commonly practiced in the region.

The two villages that report predation by wild animals to be a major problem had both suffered recent clusters of attacks on cattle by tiger. Both villages free grazed cattle within the tiger reserve buffer zone. Large predators were discussed in many other villages, particularly by older men. Younger participants usually asserted that the problem is largely historical. In each of these villages further questioning revealed that it had been some time since the last predator attack. The perceived risk of large predator attack was lower than had been expected, given that there are between 400 and 600 livestock reported to be killed by wild carnivores annually in the KTR region, and free grazing of cattle close to the core zone appears to be a major risk factor for predation [7]. Reducing ‘free grazing’ of livestock within the forest may result in less predator- livestock conflict and less degradation of the forest due to damage by livestock. This, combined with increased employment for local people in the ecotourism sector may assist conservation efforts. Alternatively decreased perception of predation of livestock could be a result the presence of larger villages and human activity degrading of the surrounding forest and affecting local carnivore populations.

Plastic ingestion by cattle was frequently discussed in village meetings and animal keepers concluded that this was an urban problem. Discussion with local para-vets confirmed this assertion, however individual survey respondents proposed plastic impaction as a major problem. Discussions during meetings sometimes indicate confusion by some animal keepers between potential and current risks to livestock. The outcome of these discussions in modifying initial opinions of individuals indicates the strength of village groups in working through problems as a community.

Paravets also identified farmer and husbandry factors as being important (Fig 6), an issue not raised by the farmers themselves, though groups often informally discussed poor access to treatment and vaccinations and the difficulties of acquiring sufficient good quality fodder. This difference in perceptions between stakeholder groups should be considered when designing education programmes.

### Current animal treatments in the KTR

Our results show a large variety of remedies in use in the KTR (Table 3 and S2–S4 Figs). Notably very few farmers discuss using pharmaceuticals, indicating a low level of usage despite availability from pharmacies in nearby towns. In the KTR pharmaceuticals appear to be mostly used through the work of para- vets. Ethno-veterinary medicine is widely practiced; however knowledge of herb lore varies greatly between villages, usually being greatest in ST settlements.

The study found poor knowledge of the causes, prevention and treatment of calf diarrhoea. Colostrum management was often poor in the KTR, similar to findings in other Indian states [23, 24, 25]. Significant amounts of colostrum are being taken for household consumption, resulting in failure of passive transfer of immunoglobulins to neonatal livestock. This suggests that simple improvements in colostrum management and the basic treatments have the potential to yield achievable and sustainable improvements.

Traditional remedies for diarrhoea often include elixir of maida bark (*Litsea glutenosa*), clay, rice husk and rice water, and these are can be effective at stopping the flow of diarrhoea, however administration of sufficient water, ideally as(homemade) electrolyte solutions to maintain hydration is also essential. Rice water has been shown to be as effective as electrolyte solution in the treatment of diarrhoea [26], however sufficient volume must be administered. Over- collection of maida bark from the forest is a cause for concern [27] and it is important that this resource and other forest products are used sustainably.

Current treatment practices for maggot wounds discussed included application of cytotoxic substances such as petrol, naphthalene (moth balls), tobacco or deltamethrin to kill maggots. This causes significant additional tissue damage resulting in prolonged healing time and increased pain and suffering to the patient, resulting in decreased productivity. These practices should be discouraged; prevention and treatment of maggot infestation can often be effected by simple cleaning of wounds and removal of eggs and larvae. Continued effort is needed to bring about attitudinal change to improve animal welfare and productivity while incurring minimal costs to the animal keeper.

Some remedies are frequently mentioned by participants (mud and coriander leaf to relieve the symptoms of FMD; maida bark, clay, and rice husk for diarrhoea; neem leaves for intestinal parasites; turmeric for skin lesions; infusions of tea and spices with sugar for mild coughs), suggesting that these treatments have a degree of efficacy. However there are also a large number of remedies used by few people. While some remedies may be effective and be worthy of further investigation, the general trend of many different treatments for a single condition often indicates the lack of an effective treatment. The widespread use of witchcraft and faith healing may also indicate the lack of access to effective treatments. There is clear need for the acquisition of knowledge of sustainable animal health practices that can be used in conjunction with traditional herbalist knowledge.

### Effectiveness of knowledge transfer activities

The results of this study show that participatory education delivered through open access meetings using simple visual aids provides effective knowledge transfer to participants (Fig 8, Table 4).

Participants displayed good visual literacy, interpreting cartoon and photographic images, discussing them and drawing conclusions. Visual illiteracy may accompany written illiteracy [13, 14], however in the KTR region participants who were unable to read readily interpreted images. Images are commonly used in India to communicate religious and public service information as well as for entertainment, which may lead to greater visual literacy among non- readers than has been previously found in some other regions Images showing an Indian context were more readily understood by participants than images taken from other regions. [14].

Open access educational meetings provided a high level of community engagement, frequently attracting large groups and a broad range of ages. Generally education programmes tend to engage more with male farmers and those from better off socio- economic groups [16]. The inclusive nature of our meetings, held in the villages, facilitated the involvement of small producers. Engaging women in education programmes in rural India can be challenging [4, 16]. The level of female participation in this education program of 27% was felt to be good, with PVE outperforming VE, despite all the paravets being male. The involvement of female paravets could further improve engagement of female animal keepers, however currently there are no women working as paravets in the KTR region. Previous veterinary studies have actively recruited equal numbers of male and female participants [10], included only male participants [12] or do not specify gender [11]; our study design aimed to highlight the challenges involved in maximising the engagement female animal keepers. Location and timing of meetings appears to be of paramount importance in maximising engagement by women. Female attendance was greatest at meetings held in the women’s home villages in the afternoon. This is because of the many roles and responsibilities of rural women; which are carried out in addition to their farming work, including: childcare, preparation of food, household tasks and additional income strategies [4]. The limitations on the availability of women to attend knowledge transfer events are highly relevant to the implementation of future programmes. Rural women in the KTR, particularly those of ST origin, have a tendency to be reserved in the presence of strangers, especially male visitors. During meetings women and men usually sat in separate groupings and in most instances women made less contributions to group discussions than men, though groups of women frequently spoke amongst themselves about the meeting content. In approximately one quarter of village meetings female voices took strong roles in the general discussion. A study in West Bengal had reported that 80% of rural female respondents cited that they were involved in making farm decisions, most commonly (33.18%) jointly with their partners [15]. Our observations in the KTR broadly concur with this level of female involvement. Maximising the engagement of women in rural education programmes is of paramount importance to improve the economic and social status of women in rural India [4] and education of women is the key route to improving the lives of children and enhancement of communities.

During meetings all participants did not contribute equally. Individuals who are well respected, usually because of age, gender or the number of cattle owned, tended to speak more frequently in group discussions, as did individuals with more vocal personalities. Other participants may acquiesce to these opinions [28] and differentiating between dominant opinions and collective opinions can be difficult [29]. While every effort was made to involve all attendees in the meeting equally, more vocal individuals have a stronger effect on the study results. However these higher status or vocal individuals will likely influence the opinions of their neighbours and the agricultural strategy of the village. Without the support of these farmers any village level intervention is unlikely to succeed [30].

The use of open access meetings presented some difficulties, both in managing the meetings and the variable return rates of participants, which in turn affected assessment meeting scores. Previous studies have paid participants to take part [12] or only included those participants that are available to attend all study visits [10]. These methodologies enhance statistical rigor, but may exclude some members of the community or result in unrealistic expectations of the effectiveness of future programmes. Our methodology provides a realistic model of the challenges of providing community education at a village level. The technique of taking a consensus answer from the focus group produced analysable data, however the nuances of the interaction between the villagers was lost [28]. Recording and transcribing group discussions would yield better understanding of knowledge and practices.

In our study VE and PVE groups performed better than the C and PM groups (Fig 8, Table 4). This differs from previous veterinary studies which found equal benefit from leaflets and community meetings [12]; measurable treatment improvements following a leaflet campaign [11]; and improvements with a leaflet campaign and no further improvement when this was supported by village meetings or videos [10].The success of these previous studies using leaflets may be the more focused subject material used: treatment of wounds in working equids, treatment of trypanosomiasis in cattle and treatment of mastitis in cattle respectively. The broader objectives of the current study may be less amenable for communication in a single leaflet. A study on HIV education in Uganda found that participants gained more benefit from meeting with a community educator than receiving a leaflet [31]. This study used six different leaflets to communicate messages on different topics, following that model the current project would need to distribute eight separate leaflets to achieve the same effect. In our study leaflets and posters were rarely observed in the villages when meetings were held four months later. This suggests that ongoing exposure to the medium, often discussed as an advantage to communication by this channel [12, 30] may be lower than hoped in the KTR region. It has been suggested that village meetings are less successful as only verbal, not visual, information is communicated [10]. The use of images as teaching aids in our study may explain the success of village meetings compared with some previous studies. Our study examined retention of knowledge transfer by participants over a period of 4 months. Leaflets have been found to provide effective retention of information in studies revisiting respondents 14 days post intervention [10, 12]. There may be an effect of improved retention of knowledge transfer over more extended periods when that knowledge transfer is delivered by face to face contact rather than through printed materials [31]. Peer and community education is susceptible to a myriad of factors: cultural; socio-economic; political; personal and technical which affect the success or failure of projects, and results are rarely consistent between regions or projects [32].

In this study distribution of Printed Materials did not provide effective knowledge transfer compared with village meetings, but the amount of time devoted by participants to the knowledge transfer activity should considered. Village meetings devoted 1–2 hours to discussing animal health issues, and participants continue to discuss matters after the meeting. Reading the leaflet required only a few minutes. A booklet taking 1–2 hours to read could deliver more information than an educational meeting, however few animal keepers would read it fully and it would be inaccessible to non- readers. Printing booklets would also incur significant costs. Printed materials do not encourage villagers to engage with each other on animal health issues or form community strategies. Educational meetings also allow flexibility for clarification of points and discussion of the local situation, however the potential contribution of printed materials to community education meetings should not be overlooked as this would engage multiple learning channels and provide information to take home for reference. Participatory village meetings yield additional benefits, bringing communities together to discuss issues increases engagement and empowerment which enables self- help group foundation, popular education and political participation [4].

It is worth noting that two villages in the VE group performed significantly less well than the others within this group (Kruskal- Wallis, P<0.001). These villages were inhabited by people of ST origin. Removing these ST villages from the statistical analysis increased the significance of the gains made by the VE group, with VE performed significantly better than all other groups (C (P = 0.0216); PM (P = 0.0361); and PVE (P = 0.0174)). However ST people represent an important demographic in the KTR and removing these villages from the analysis would underestimate the challenges encountered in providing farmer education in the KTR region.

There was considerable variation in the educational gains made on different conditions. Conditions which were readily identified by farmers, such as diarrhoea, maggots and FMD made the most notable gains. Less easily recognised conditions, such as intestinal worms and lantana toxicity, showed little improvement. The greatest gains are found in conditions which are easily recognised but poorly understood (diarrhoea), however traditional beliefs and cultural factors can pose a significant barrier to change (colostrum consumption, diarrhoea and maggots). In some cases only part of the intended message was retained by participants. For example, home remedies for the infestation of wounds by maggots frequently include the application of caustic or cytotoxic substances to wounds, the education programme discouraged this in favour of frequent wound cleaning, at the second meeting VE and PVE groups often describe careful wound cleaning followed by the application of cytotoxic substances.

Training of paravets as educators is a realistic and sustainable way to contact large numbers animal keepers. Training paravets also has wider benefits, paravets are active within communities treating animals and will have on-going discussions that broaden and reinforcing animal health messages. Education programmes also raise the status of the para- vet within the village. When possible paravets also should to be supplied with appropriate teaching aids. These must be clear, well thought out and inexpensive to produce. Training of paravets as educators by practicing veterinarians also provides an opportunity for the paravets to increase their own veterinary knowledge.

## Conclusions

In the KTR region there is both a need and a desire for improved animal health knowledge. Participatory village meetings are an inclusive method of sharing knowledge with the potential to meet this need and bring community members together to form common strategies for development Paravets working as community educators have the potential to deliver animal health knowledge transfer rapidly and in a sustainable manner, but they should be supported with adequate training and teaching aids. Village meetings should be carefully timed, with attention paid to the local situation, to maximise the involvement of all members of the community, particularly women. There is variation in the gains made through education on different subjects, in this study diarrhoea, maggot wounds and control of infectious disease (FMD) appear to offer the greatest benefits for the efforts applied. Education of animal keepers on the importance of adequate colostrum consumption by neonates to prevent disease via maternally derived antibody should be singled out as offering great potential gains at low cost.

Further work is required to refine the processes of design and dissemination of knowledge transfer programmes, however the training of paravets has the potential to yield rapid, sustainable benefits to rural people.

## Supporting information

S1 Fig Images to stimulate discussion. Used at second (assessed) visit.
(TIF)

S2 Fig Current treatments in use in the KTR. FMD and maggot wounds.
(TIF)

S3 Fig Current treatments in use in the KTR. Diarrhoea.
(TIF)

S4 Fig Current treatments in use in the KTR. BRD and intestinal parasites.
(TIF)

S5 Fig Group scores by topic. (TIF)

S6 Fig Group scores by question type. (TIF)

S1 Table Local animal health terms used in the KTR. (TIF)

S2 Table Full list of assessed meeting scores. Includes breakdown by topic.
(TIF)

S3 Table Full Kruskal- Wallis table, including breakdown by topic. (TIF)

S4 Table Full Kruskal- Wallis table, including breakdown by question type. (TIF)

S1 Text Individual animal keeper questionnaire. Pre- intervention.
(TIF)

S2 Text Paravet (Gau Sewek) questionnaire. (TIF)

S3 Text Second visit questionnaire part 1. Used in conjunction with S3 Fig images to generate assessment scores.
(TIF)

S4 Text Second visit questionnaire part 2. (TIF)
