# Correction: Delivery and evaluation of participatory education for animal keepers led by veterinarians and para-veterinarians around the Kanha Tiger Reserve, Madhya Pradesh, India

**DOI:** 10.1371/journal.pone.0203299

**Published:** 2018-08-27

**Authors:** Andy Hopker, Naveen Pandey, Aniruddha Dhamorikar, Sophie Hopker, Pradeep Gautam, Subash Pandey, Sharad Kumar, Narendra Rahangadale, Prakash Mehta, Rebecca Marsland, Neil Sargison

The following information is missing from the Funding section: The work was conducted using the facilities of the Roslin Institute funded by the BBSRC. NS is supported by BBSRC through the Institute Strategic Programme funding (BB/J004235/1 and BB/P013740/1).
