# Supplementary figures and images for: Delivery and evaluation of participatory education for animal keepers led by veterinarians and para-veterinarians around the Kanha Tiger Reserve, Madhya Pradesh, India
Source: PLoS One. 2018 Aug 2;13(8):e0200999. doi: 10.1371/journal.pone.0200999 (PMC6071983; doi:10.1371/journal.pone.0200999)

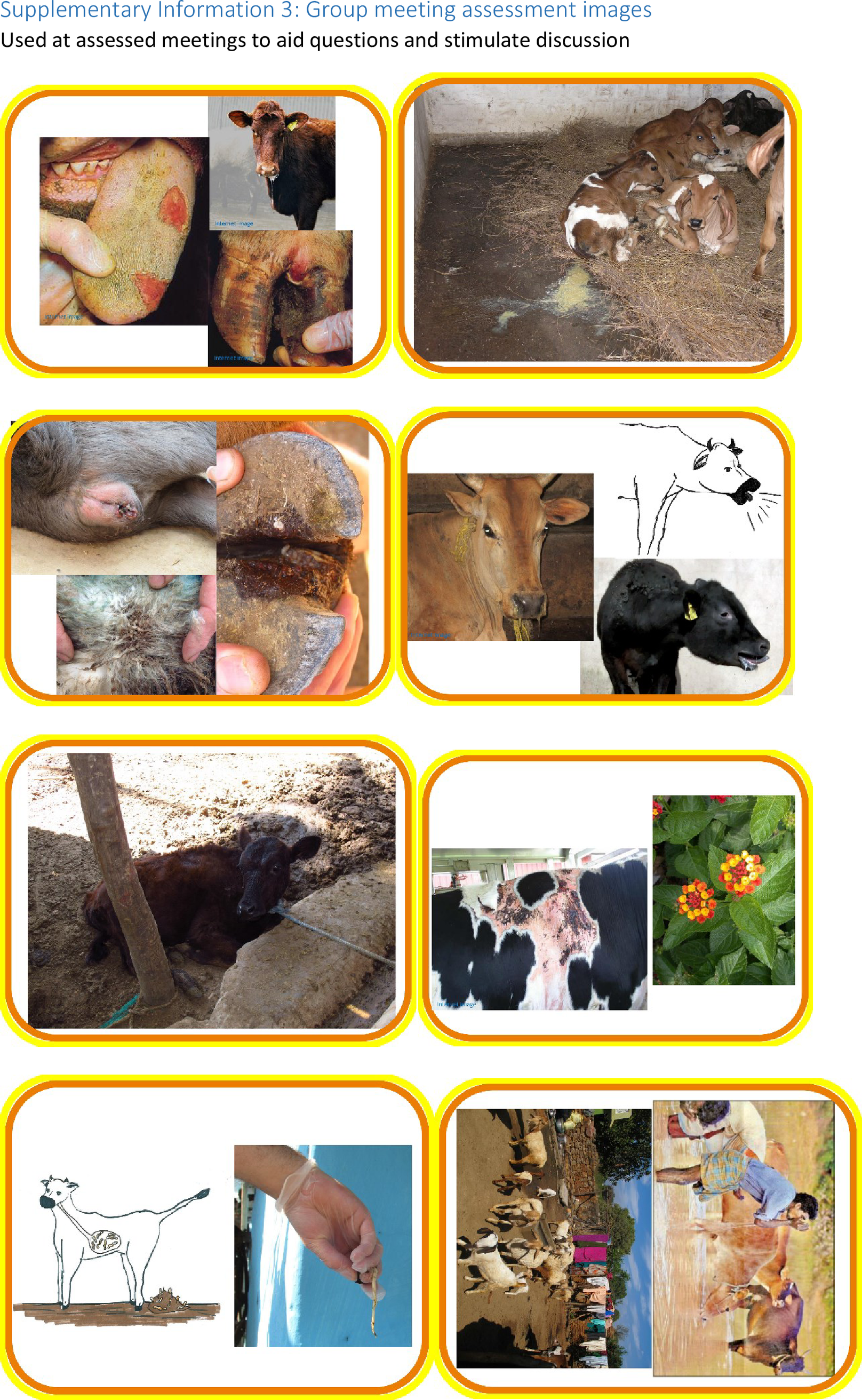

Supplement: S1 Fig — Used at second (assessed) visit. (TIF) [file pone.0200999.s001.tif]

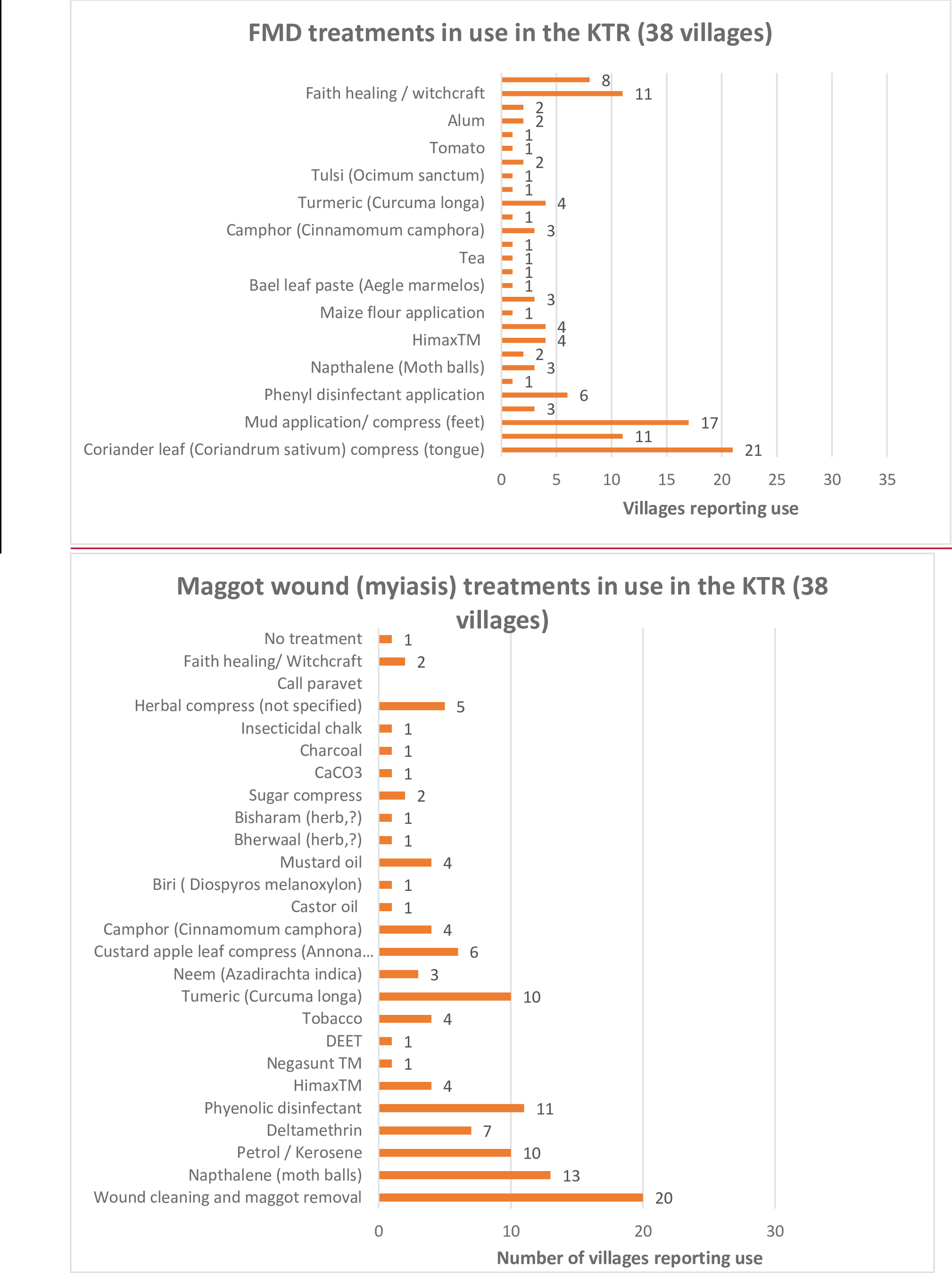

Supplement: S2 Fig — FMD and maggot wounds. (TIF) [file pone.0200999.s002.tif]

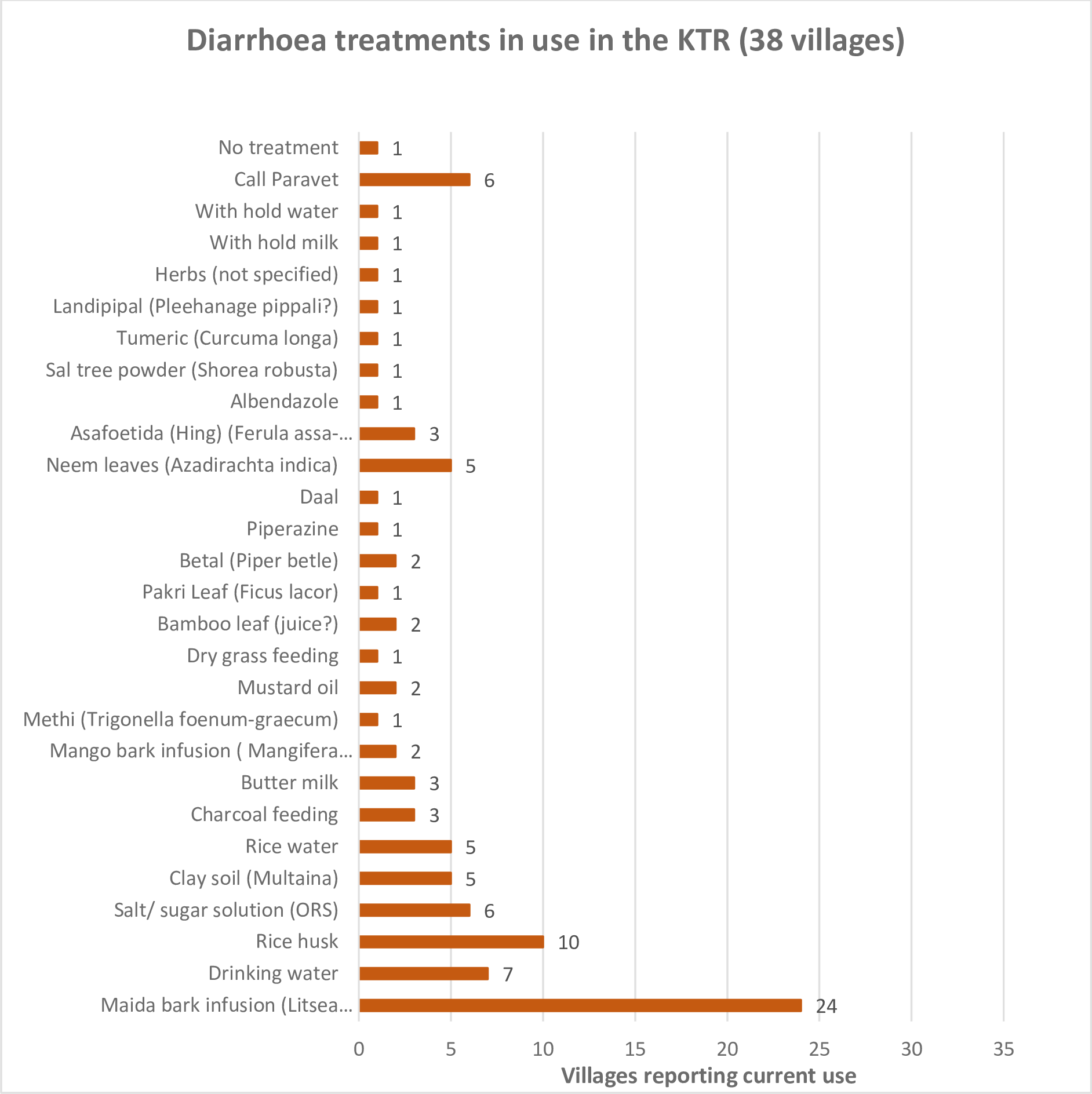

Supplement: S3 Fig — Diarrhoea. (TIF) [file pone.0200999.s003.tif]

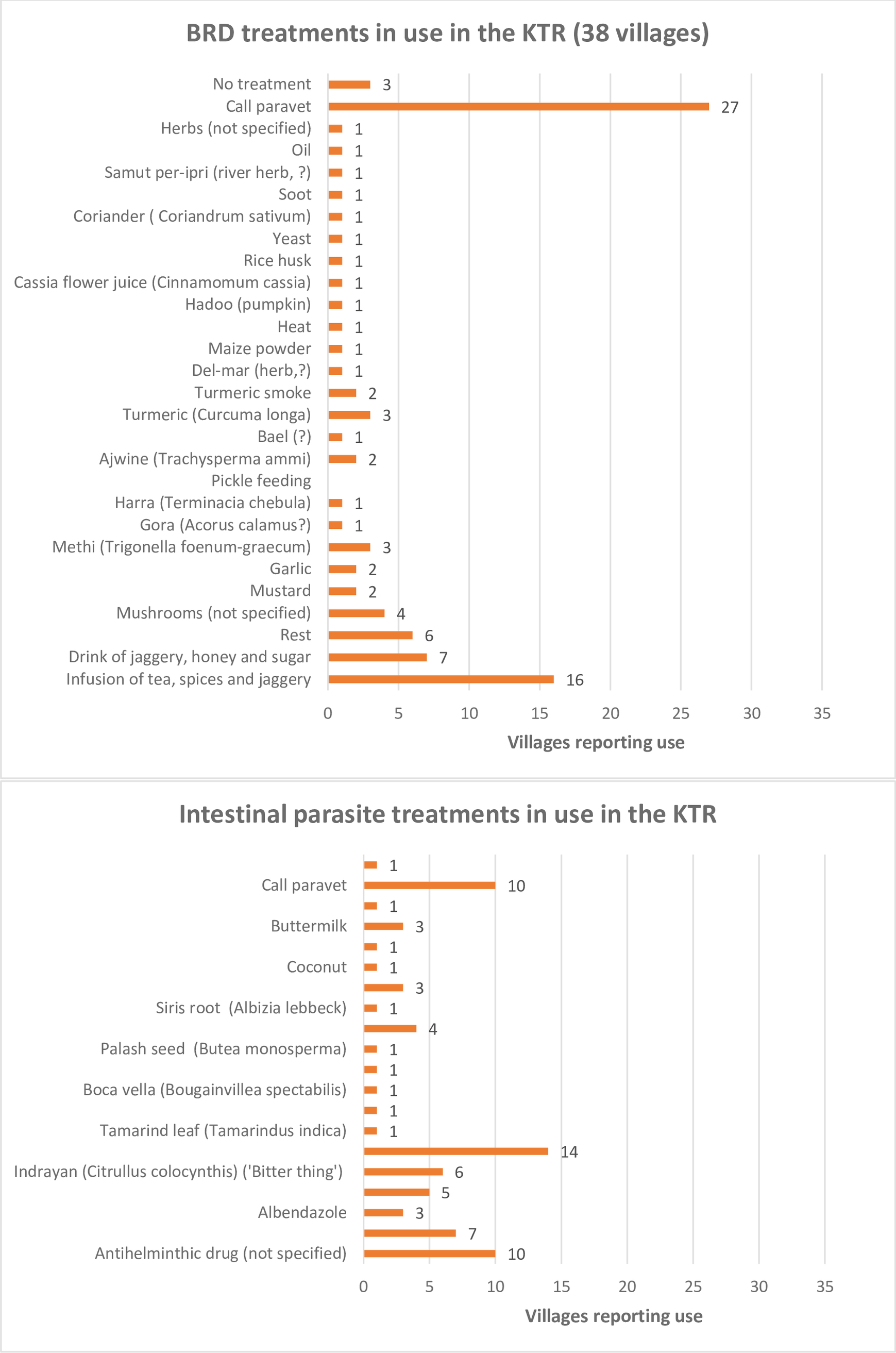

Supplement: S4 Fig — BRD and intestinal parasites. (TIF) [file pone.0200999.s004.tif]

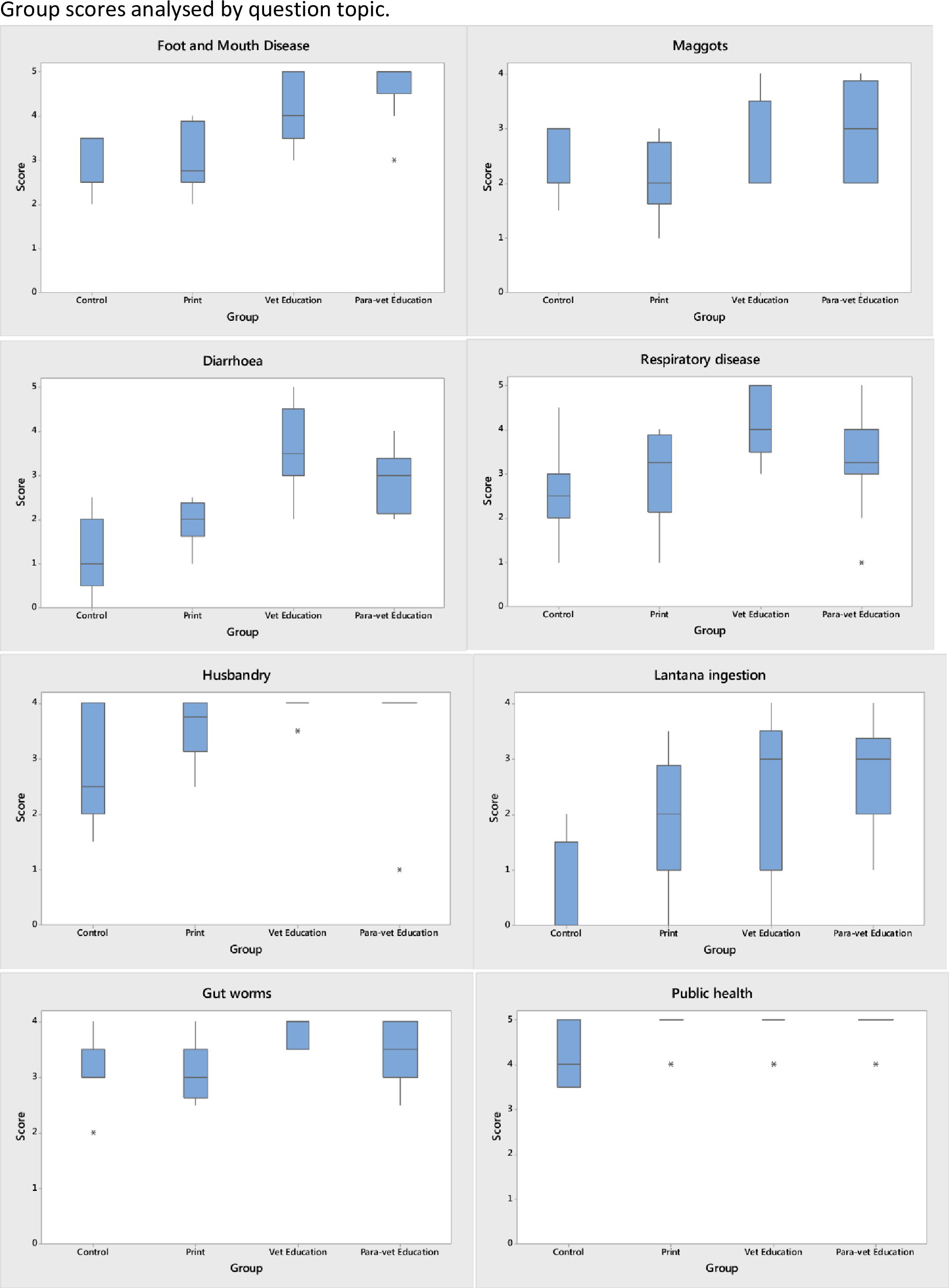

Supplement: S5 Fig — (TIF) [file pone.0200999.s005.tif]

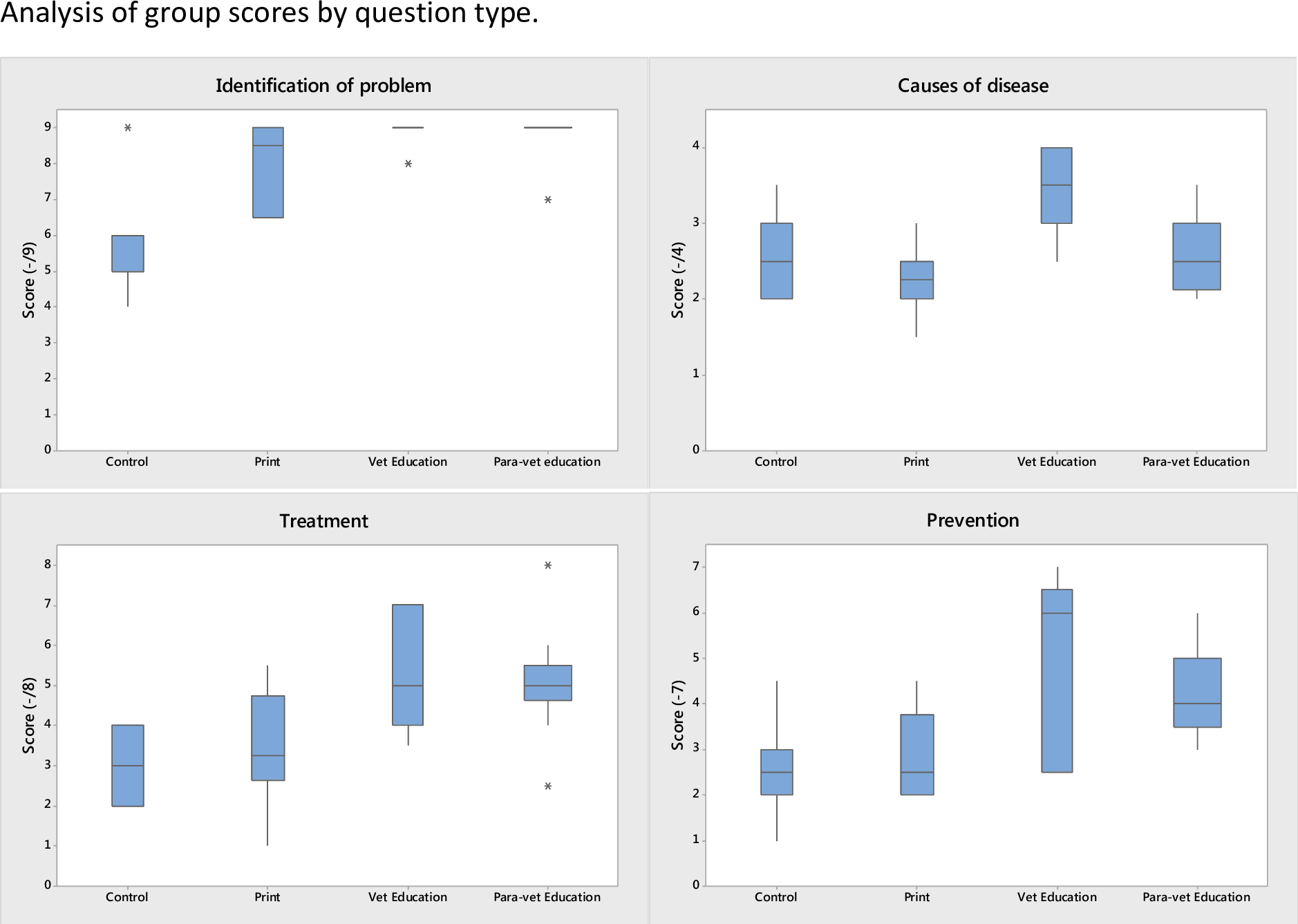

Supplement: S6 Fig — (TIF) [file pone.0200999.s006.tif]

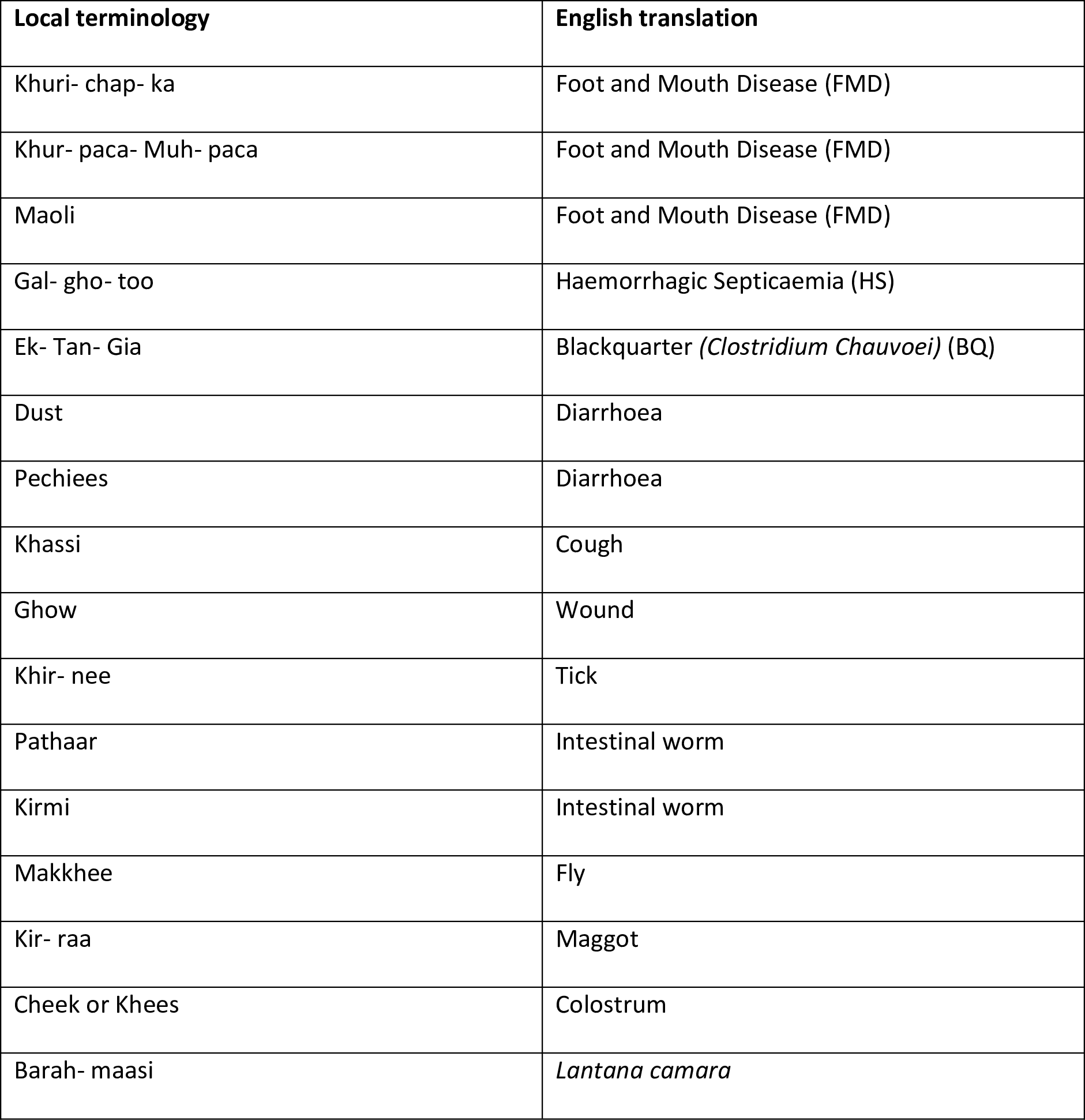

Supplement: S1 Table — (TIF) [file pone.0200999.s007.tif]

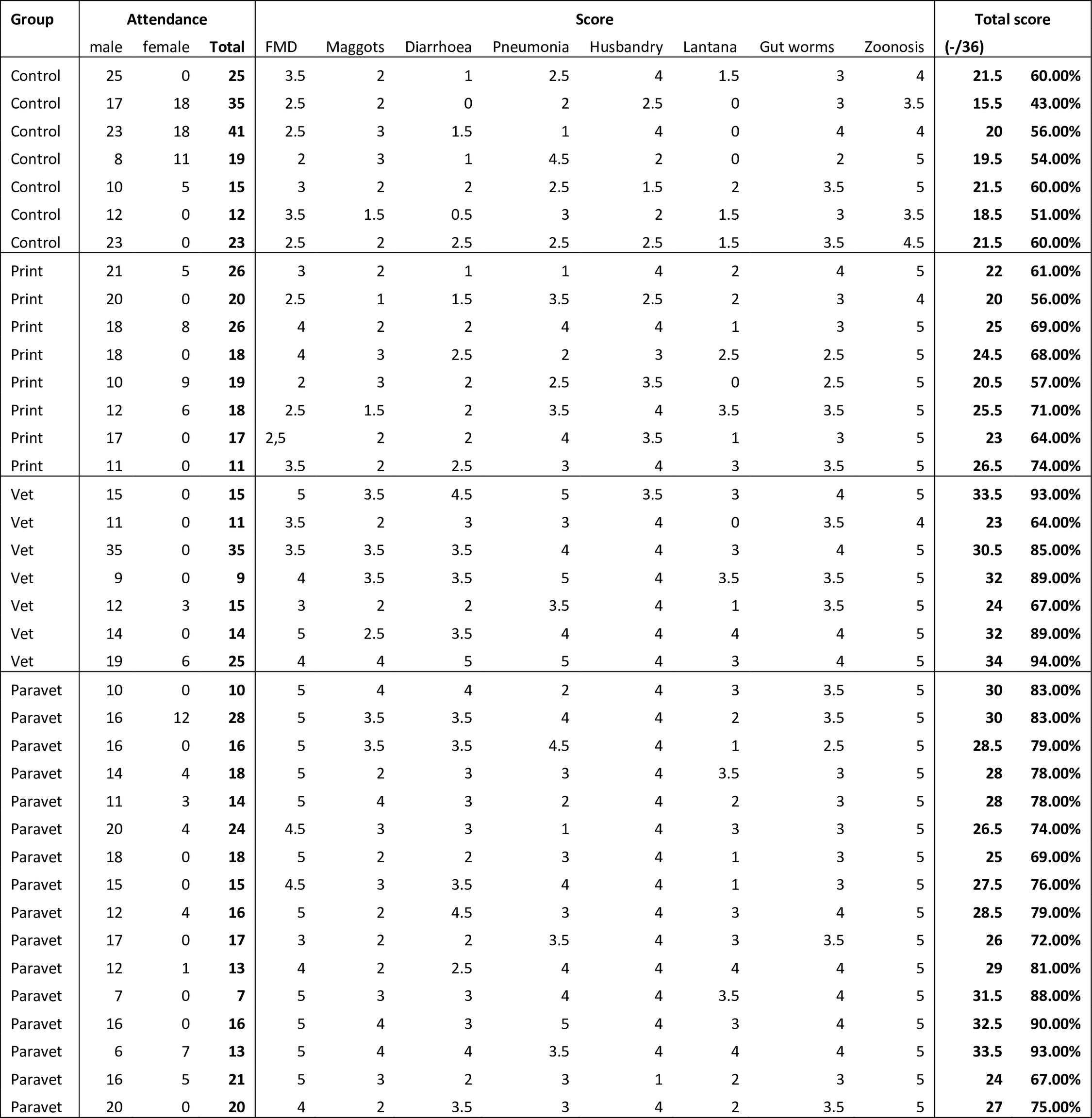

Supplement: S2 Table — Includes breakdown by topic. (TIF) [file pone.0200999.s008.tif]

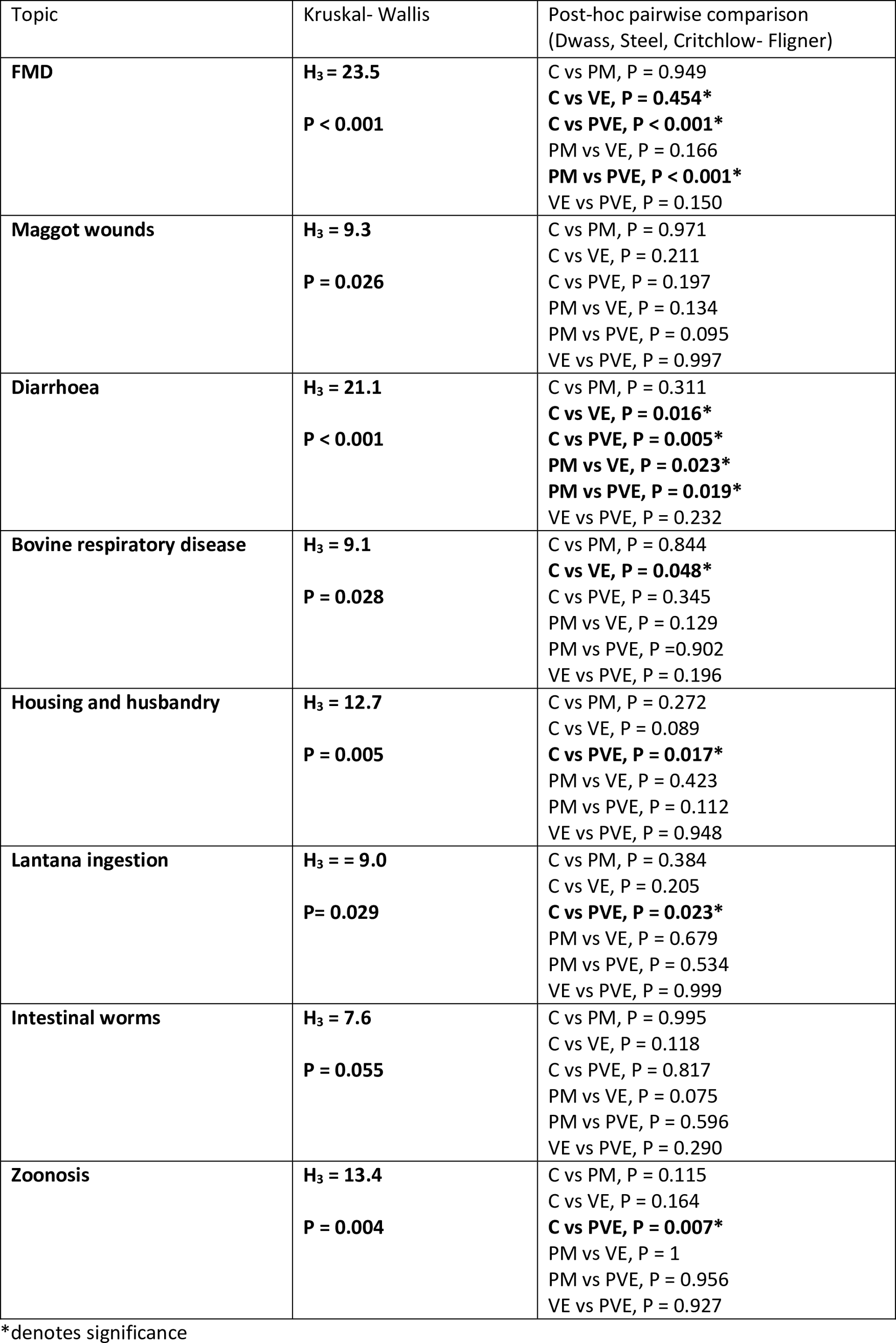

Supplement: S3 Table — (TIF) [file pone.0200999.s009.tif]

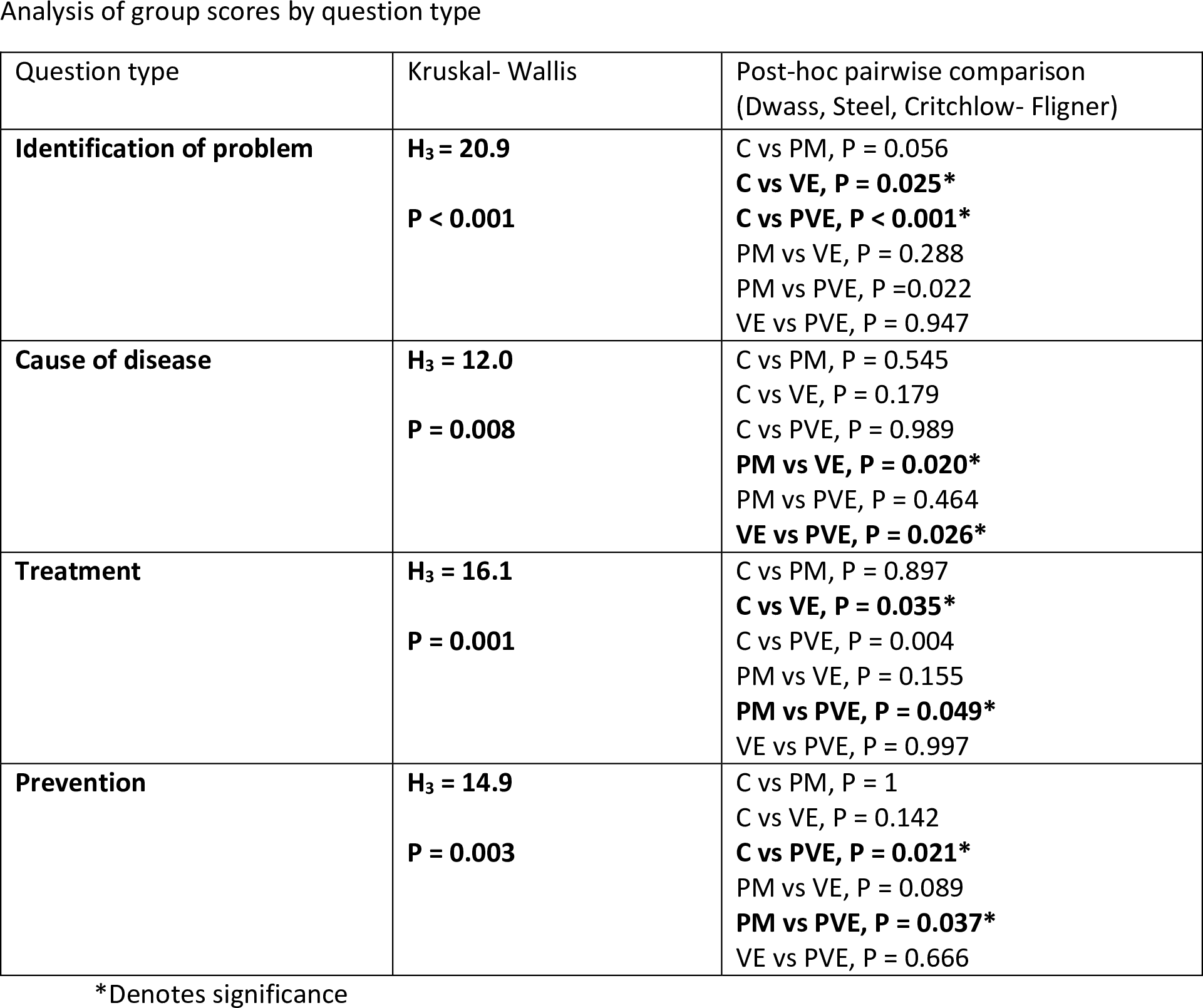

Supplement: S4 Table — (TIF) [file pone.0200999.s010.tif]

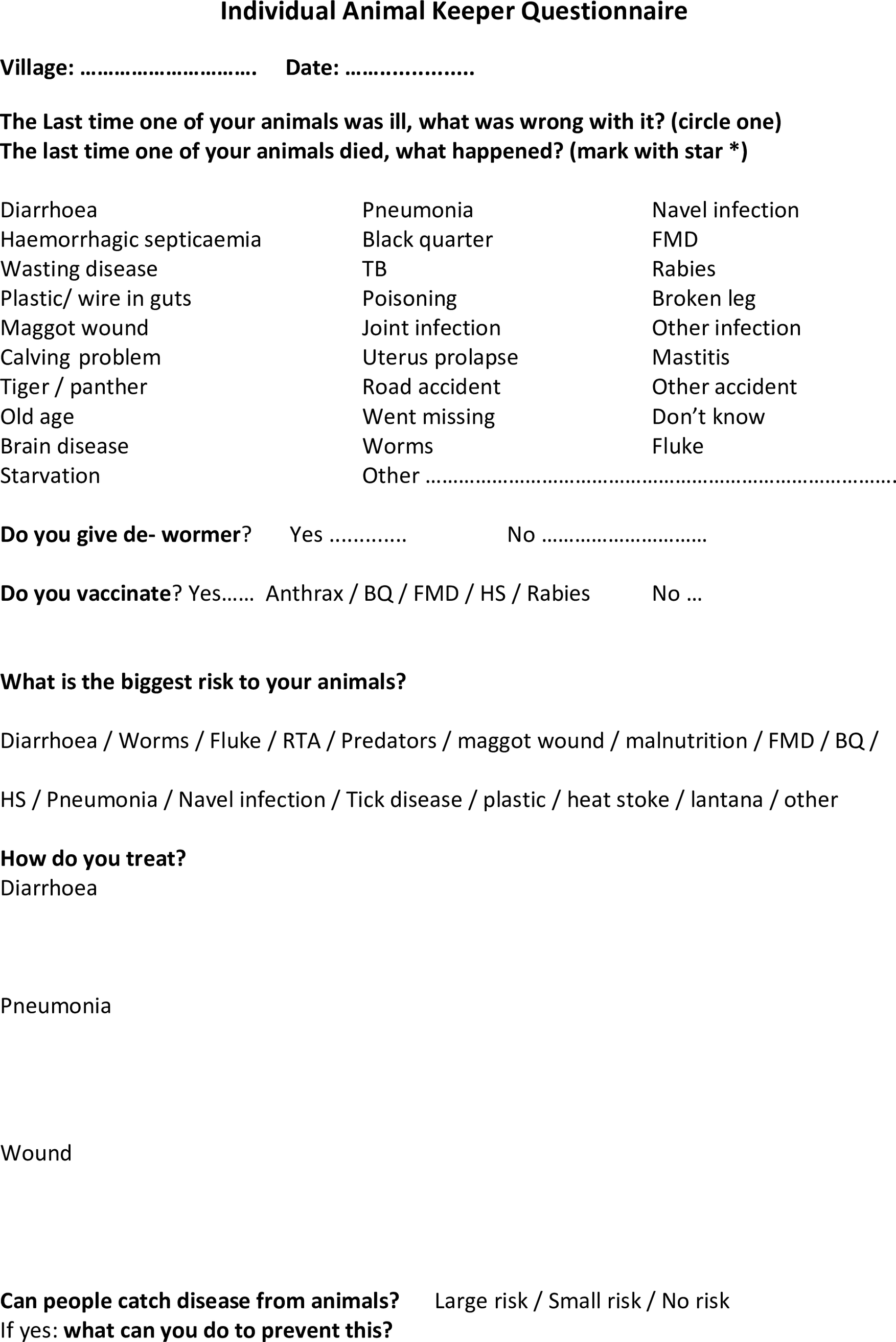

Supplement: S1 Text — Pre- intervention. (TIF) [file pone.0200999.s011.tif]

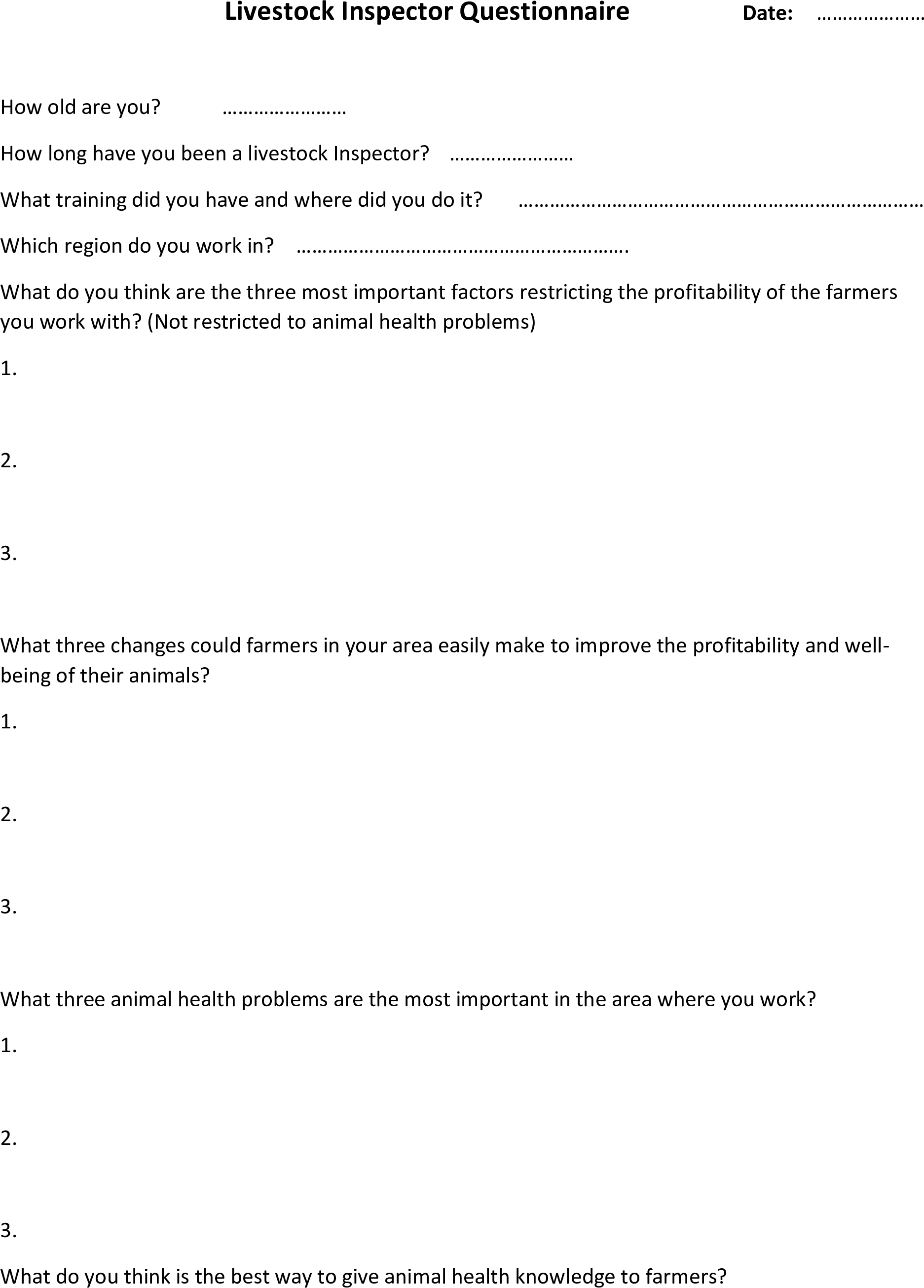

Supplement: S2 Text — (TIF) [file pone.0200999.s012.tif]

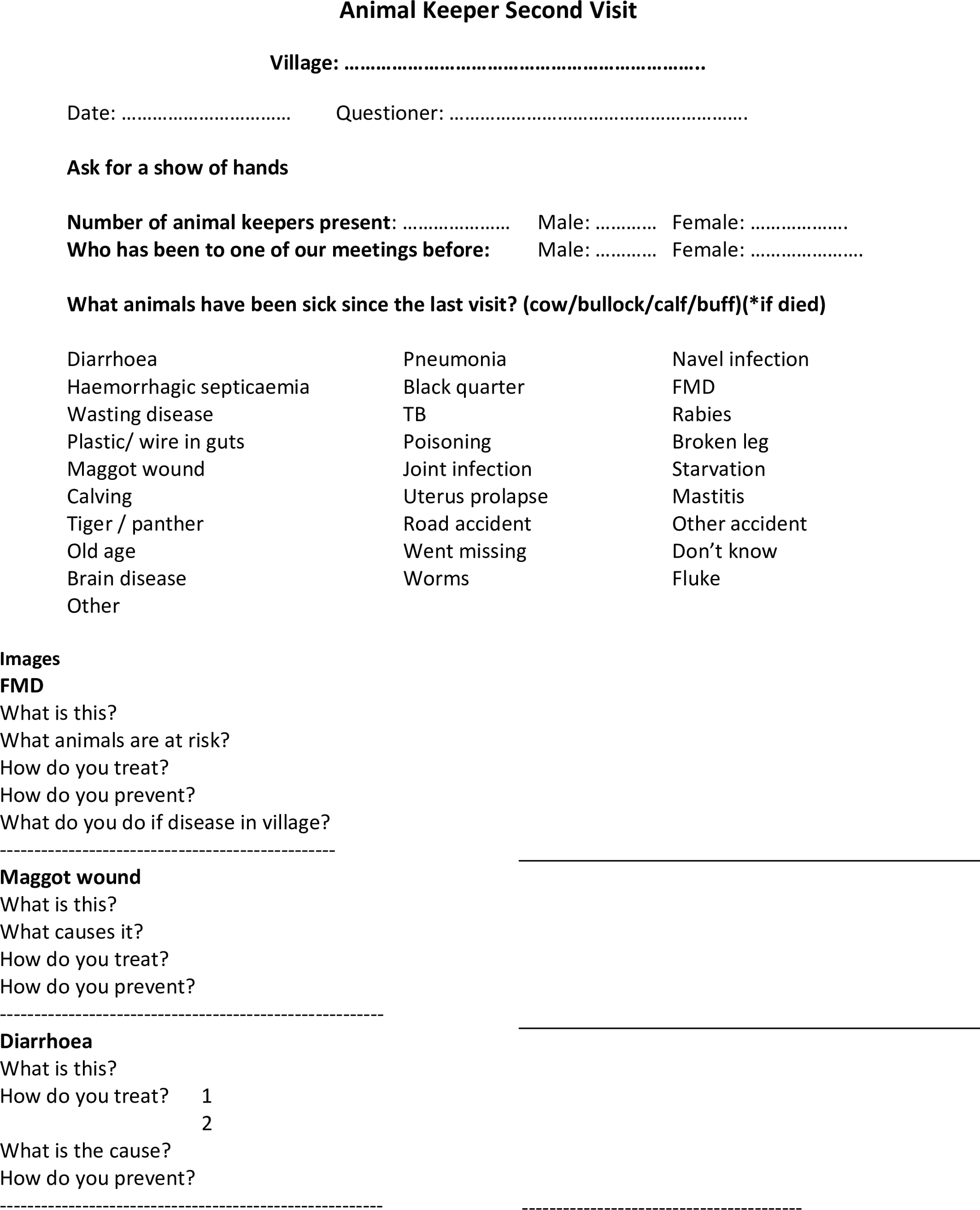

Supplement: S3 Text — Used in conjunction with S3 Fig images to generate assessment scores. (TIF) [file pone.0200999.s013.tif]

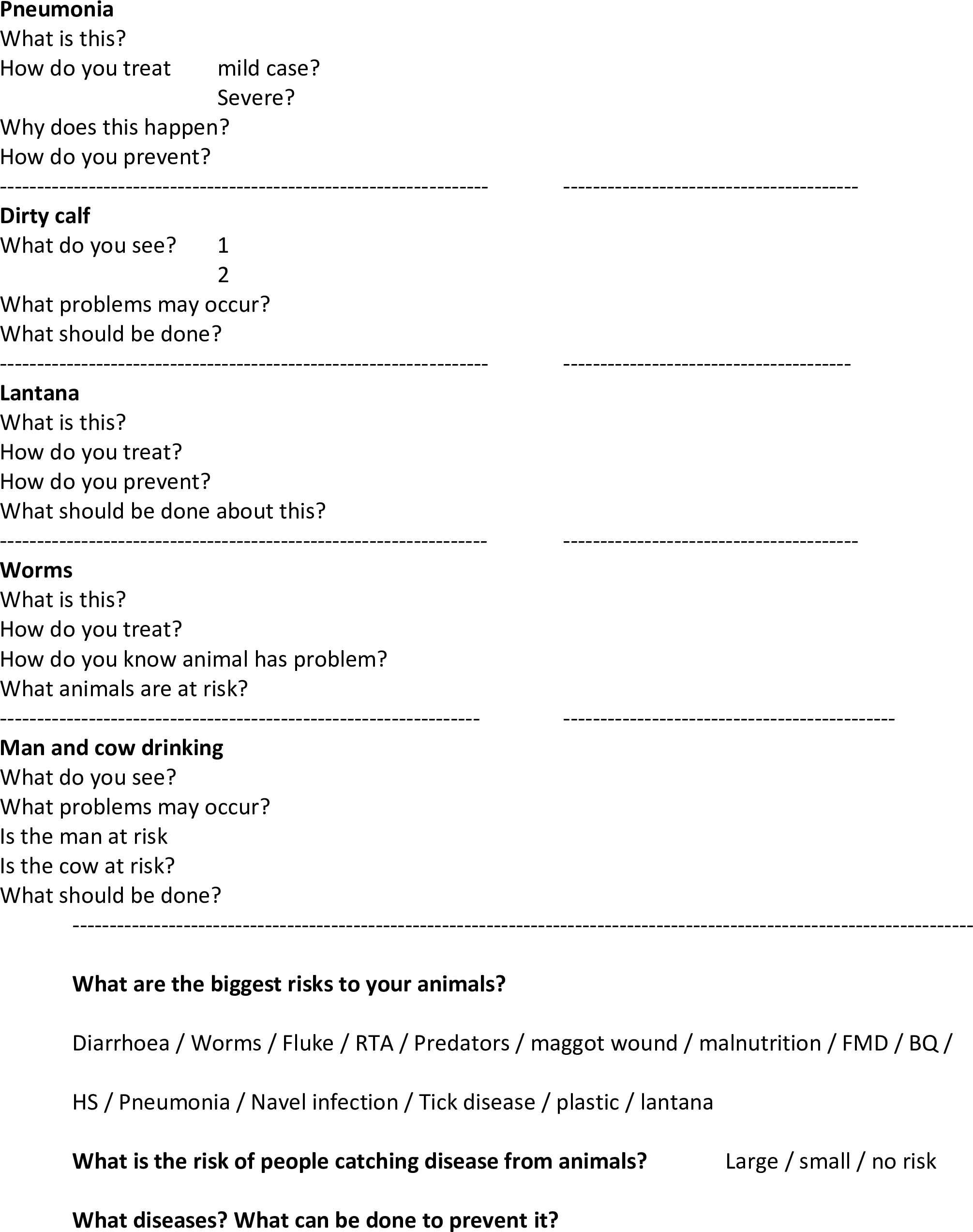

Supplement: S4 Text — (TIF) [file pone.0200999.s014.tif]
